# Thermal Transformation of Selected Non-Steroidal Anti-Inflammatory Drugs Trapped in Silica Beads via Amine-Functionalised Silica Gel

**DOI:** 10.3390/ma19194193

**Published:** 2026-09-30

**Authors:** Dariusz Sternik, Andrzej Sienkiewicz, Marek Gorgol, Joanna Lupa, Radosław Zaleski, Agnieszka Kierys

**Affiliations:** 1 Department of Physical Chemistry, Institute of Chemical Sciences, Maria Curie-Sklodowska University, M. Curie-Sklodowska Sq. 3, 20-031 Lublin, Poland; andrzej.sienkiewicz@umcs.pl (A.S.); joanna.lupa@mail.umcs.pl (J.L.); 2 Department of Materials Physics, Institute of Physics, Maria Curie-Sklodowska University, M. Curie-Sklodowska Sq. 1, 20-031 Lublin, Poland; marek.gorgol@umcs.pl (M.G.); radek@zaleski.umcs.pl (R.Z.); 3 Doctoral School of Quantitative and Natural Sciences, Chemical Sciences, Maria Curie-Sklodowska University, Weteranów St. 18, 20-400 Lublin, Poland

**Keywords:** TG, DSC, naproxen, sodium ibuprofen, TG/FTIR/MS, silica pellets, porous silica, API, positron annihilation lifetime spectroscopy (PALS)

## Abstract

**Highlights:**

**What are the main findings?**

- Silica confinement thermally stabilises naproxen, unlike sodium ibuprofen.
- Signals attributable to volatile ibuprofen were detected during heating of sodium ibuprofen in helium and, less prominently, in synthetic air.
- The hybrid amine-functionalised silica phase stabilises both dispersions in helium and appears to contribute substantially to oxidation in air.

**What are the implications of the main findings?**

- Carrier–drug interactions must be tailored to the physicochemical form of the API.
- Drug loading alone does not imply a protective effect of the carrier.
- Thermal behaviour should be an essential part of the carrier design process.

**Abstract:**

This study presents solid dispersions of naproxen and sodium ibuprofen in porous SiO_2_ pellets (manufactured via the hard template method) before and after additional phase manufacture from amine-functionalised silica gel. Nanoscopic pore evolution of the investigated materials was examined at all stages of synthesis using positron annihilation lifetime spectroscopy (PALS). The TG–DSC–FTIR/MS in He and synthetic air provided information on the systems’ thermal transformations. PALS showed that both drugs preferentially occupied smaller mesopores in SiO_2_ pellets. Thermal analysis revealed distinct atmosphere- and drug-dependent behaviour. Silica confinement delayed naproxen’s thermal degradation but decreased sodium ibuprofen stability. Interestingly, thermal decomposition of sodium ibuprofen most likely also occurred through the evolution of volatile ibuprofen, as signals assigned to it were detected in both atmospheres, although less extensively in air. Encapsulation of naproxen in the investigated systems made it more resistant to high temperature, whereas for sodium ibuprofen the opposite tendency was observed. The amine-functionalized silica phase increased the overall thermal stability of both dispersions in helium, whereas oxidation in synthetic air seemed to be governed mainly by the NH_2_-SiO_2_ phase degradation.

## 1. Introduction

Many efforts have been made to develop new systems that can control both the rate and period of active pharmaceutical ingredient (API) delivery, as well as the place of their administration. In such systems, the carrier is of fundamental importance, as it influences solubility, stability, bioavailability, and targetability of the API, as well as protecting it from premature degradation and improving patient acceptability and adherence [1,2,3,4,5,6]. Amorphous silica is an attractive carrier for active pharmaceutical ingredients. Approved as a food additive E551 in the European Union (EU) [7] and permitted for use as an anticaking agent in foods under specified conditions by the US Food and Drug Administration (FDA) [8], it is widely used in solid dosage forms such as tablets as a glidant or lubricant [9,10]. Beyond conventional uses, silica finds applications in, for instance, implantable therapeutic systems [11,12,13,14] and in bone [15,16] and tooth [17,18] repair. Functionalised mesoporous silica nanoparticles show great promise for developing targeted bio-imaging contrast agents, including optical imaging, magnetic resonance imaging (MRI), positron emission tomography (PET), computed tomography (CT), ultrasound imaging, and multimodal imaging for the early diagnosis of various illnesses [19,20,21]. No wonder that silica and/or silica-based materials are extensively investigated for encapsulation of drugs for their controlled delivery or release [22,23,24,25].

This study investigates drug-loaded silica materials in which porous silica pellets serve as the API carrier, and the amine-functionalized silica gel phase (the NH_2_-SiO_2_ phase) acts as a modifier. Silica pellets were synthesised via the hard-template method, using polymer microspheres as a template and ammonia supplied in the gas phase as a catalyst to transform the silica precursor present in the polymer pores [26,27,28,29]. Sodium ibuprofen and naproxen, commonly used in the pharmaceutical industry as over-the-counter drugs and extensively studied as model carboxylic drugs, were selected for the study. The NH_2_-SiO_2_ phase composition was selected based on reports that 3-aminopropyl functional groups, grafted onto the carrier surface prior to loading with naproxen and sodium ibuprofen, can influence their release rate [1,30,31,32,33,34,35]. Silica deposition after drug loading has previously been demonstrated in polymer-based systems [30,31,36]. Post-loading functionalisation with TEOS/APTES was also reported for diclofenac sodium dispersions in polymer and polymer–mesoporous silica carriers [32,37]. These earlier investigations included PALS characterisation [37,38] and, for ibuprofen sodium-loaded polymer–silica composites, DSC and TG–FTIR/MS analysis in helium [36]. Building on established routes to porous silica pellets [28,29], the present work investigates drug-loaded silica carriers obtained after removal of the polymer template. Its original contribution is a comparative assessment of how drug identity and subsequent modification with an amine-functionalised silica phase affect free-volume structure and thermal behaviour in helium and synthetic air. This assessment combines PALS with TG–DSC–FTIR/MS and compares neat naproxen and sodium ibuprofen, their dispersions in silica pellets and the subsequently functionalised materials. The use of a silica carrier enables these thermal transformations to be investigated without additional decomposition of a polymeric carrier. The conditions of the amine-functionalised silica phase manufacture were selected to minimise loss of the active substances during preparation. Gelation of silica and hybrid silica precursors (i.e., tetraethoxysilane (TEOS) and (3-aminopropyl)triethoxysilane (APTES)) was carried out in the presence of water and ammonia vapours at autogenous pressure and at ambient temperature [29,31,32,36,38], leading to the formation of an amino-functionalized silica phase, analogous to amino-functionalized groups attached to mesoporous silica nanoparticles or mica [39,40,41]. This approach yields multicomponent systems containing a silica phase functionalized with amine groups—potentially extending into the depth of the available pore network. This study aims to precisely characterise their thermal stability and decomposition, as well as naproxen and sodium ibuprofen and their solid dispersions in porous silica pellets. Analyses were conducted in both oxidative and non-oxidative atmospheres using thermogravimetric (TG) measurements and differential scanning calorimetry (DSC). Simultaneously, volatile species evolving during heating in all investigated systems were detected and identified with Fourier-transform infrared spectroscopy (FTIR) and mass spectrometry (MS). The analysis and discussion focus on the thermal stability of the NH_2_-SiO_2_ phase composed of amine-functionalized silica gel, as it may play a crucial role in the storage and stability of solid dispersions of APIs in porous silica pellets. An additional goal of this study was to present the porosity changes before and after formation of the amine-functionalized silica gel phase in solid dispersions of API in porous silica pellets at the nanoscopic level. The ability to probe various free volumes, including closed pores and nanopores, and their changes is crucial for designing such systems, where the NH_2_-SiO_2_ phase-manufacturing method favours the formation of these types of voids. Because classical methods based on gas adsorption and desorption measurements do not provide information about closed pores and are limited in examining nanopores, positron annihilation lifetime spectroscopy (PALS), a highly sensitive tool for probing various free volumes [37,38,42,43], including closed ones, was used to study these materials.

## 2. Materials and Methods

### 2.1. Materials

(3-aminopropyl)triethoxysilane (APTES; 98%, Merck, Darmstadt, Germany) and tetraethoxysilane (TEOS; 98%, Merck, Darmstadt, Germany), serving as the silica sources and model drugs, i.e., sodium ibuprofen (IbuNa; sodium 2-(4-isobutylphenyl)propanoate, ≥98%, CAS No: 31121-93-4) and naproxen (Nap; (*S*)-(+)-6- methoxy-α-methyl-2-naphthaleneacetic acid, 98%, CAS No: 22204-53-1) were supplied by Sigma-Aldrich (St. Louis, MO, USA) and used as received. Macroporous resin Amberlite^®^XAD7HP (ROHM & HAAS, Philadelphia, PA, USA, fraction of beads with an average diameter of (595 ± 178) μm) was employed as a hard template for the silica pellet synthesis. Ammonia solution (NH_4_OH, 25%) and ethanol (96%), p.a. grade, supplied by POCH, Gliwice, Poland, were used without further purification.

### 2.2. Materials Preparation

#### 2.2.1. Preparation of Silica Pellets (SiO_2_)

**Scheme 1:**
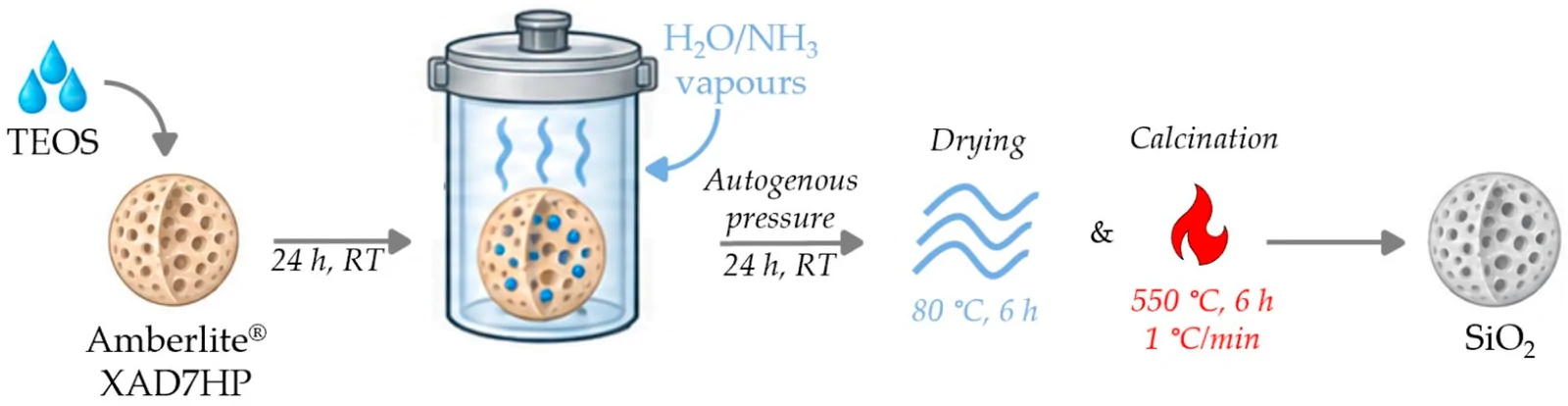
Schematic representation of the silica pellets (SiO_2_) synthesis via the hard-template method, using polymer microspheres Amberlite^®^XAD7HP as a template and ammonia supplied in the gas phase as a catalyst to transform the silica precursor (TEOS).

Silica pellets were prepared according to the detailed procedure given in Refs. [28,29]. The dry Amberlite^®^XAD7HP beads were immersed in TEOS, whose amount was adjusted so that the beads were completely wetted, with no TEOS excess outside the beads. The Amberlite^®^XAD7HP beads imbibed TEOS and increased their volume. Resin beads saturated with TEOS were aged at room temperature in a closed, airtight container for 24 h. Next, they were transferred into a vacuum desiccator and subjected to a mixture of water and ammonia (200 mL of freshly prepared 6.68 mol L^−1^ NH_4_OH) vapours at autogenous pressure and room temperature for 1 day to facilitate the transformation of TEOS into silica gel. Afterwards, the composite was dried at 80 °C for 6 h and then calcined at 550 °C for 6 h in air with a heating rate of 1 °C min^−1^. The resulting silica pellets were labelled SiO_2_. Scheme 1 illustrates the synthesis procedure for silica pellets (SiO_2_).

#### 2.2.2. Drug Loading Procedure

**Scheme 2:**
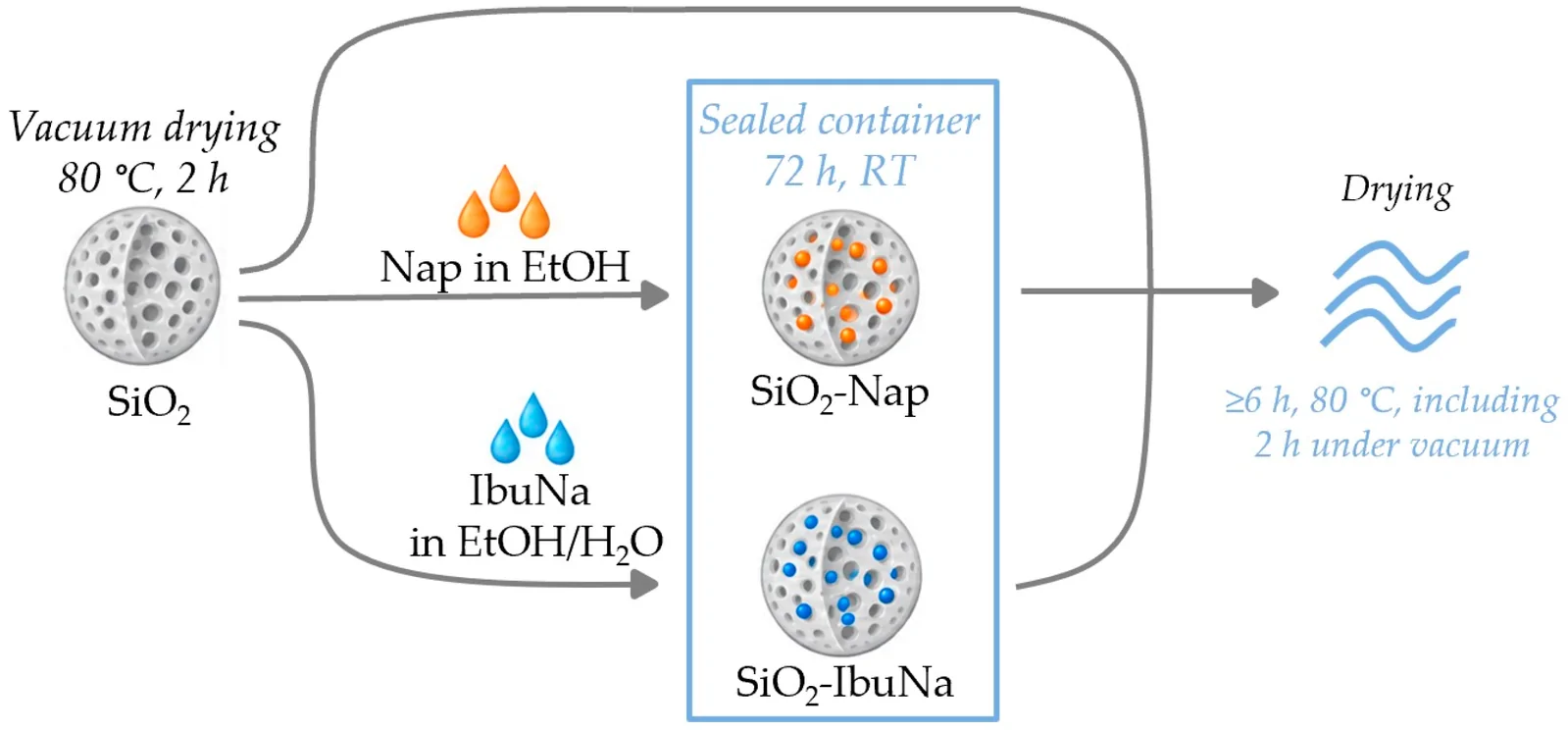
Schematic representation of the synthesis of solid dispersions of drugs (SiO_2_-Nap and SiO_2_-IbuNa) *via* incipient wetness impregnation.

The drugs were introduced into previously dried silica pellets (80 °C under vacuum for 2 h) by the method considered to be incipient wetness impregnation, as the volume of the drug solutions of appropriate concentration was equal to or only slightly higher than the pore volume of the silica support. Naproxen was dissolved in anhydrous ethanol (1.0 g Nap per 4.9 g of EtOH/17% wt). In contrast, sodium ibuprofen was dissolved in a binary mixture of ethanol (EtOH) and water, with a molar fraction of EtOH equal to 0.7 (1 g of IbuNa per 6.9 g of the solvent mixture/12.65% wt). The drug solutions prepared by ultrasonic dissolution were added to the silica pellets. SiO_2_ saturated with the drug solution was kept at room temperature in a closed, airtight container for 72 h. Afterwards, the samples were dried for at least 6 h, including 2 h, in a vacuum dryer at 80 °C. The dry solid dispersion of naproxen was labelled SiO_2_-Nap, whereas SiO_2_-IbuNa refers to the SiO_2_ with sodium ibuprofen. Scheme 2 illustrates the synthesis of SiO_2_-Nap and SiO_2_-IbuNa.

Drug loadings were estimated from TG measurements in synthetic air using the cumulative mass losses up to 800 °C. The calculation accounted for the 1% mass loss of the carrier in the form of vacuum-dried SiO_2_ pellets. Assuming additive mass losses, the drug mass fraction (ω_drug_) was calculated as

ω_drug_ = (L_solid dispersion_ − L_SiO2_)/(L_drug_ − L_SiO2_), (1)

where L_solid dispersion_, L_SiO2_ and L_drug_ denote the fractional mass losses of the solid dispersions of drugs (SiO_2_-Nap and SiO_2_-IbuNa, respectively), vacuum-dried SiO_2_ pellets and neat naproxen and sodium ibuprofen, respectively. This yielded approximate loadings of 9 wt% for SiO_2_–Nap and 23 wt% for SiO_2_–IbuNa. The naproxen estimate assumes essentially complete removal of drug-derived species by 800 °C, whereas the sodium ibuprofen estimate assumes that its residual fraction is unchanged by incorporation into silica.

#### 2.2.3. Multicomponent Systems Manufacture

**Scheme 3:**
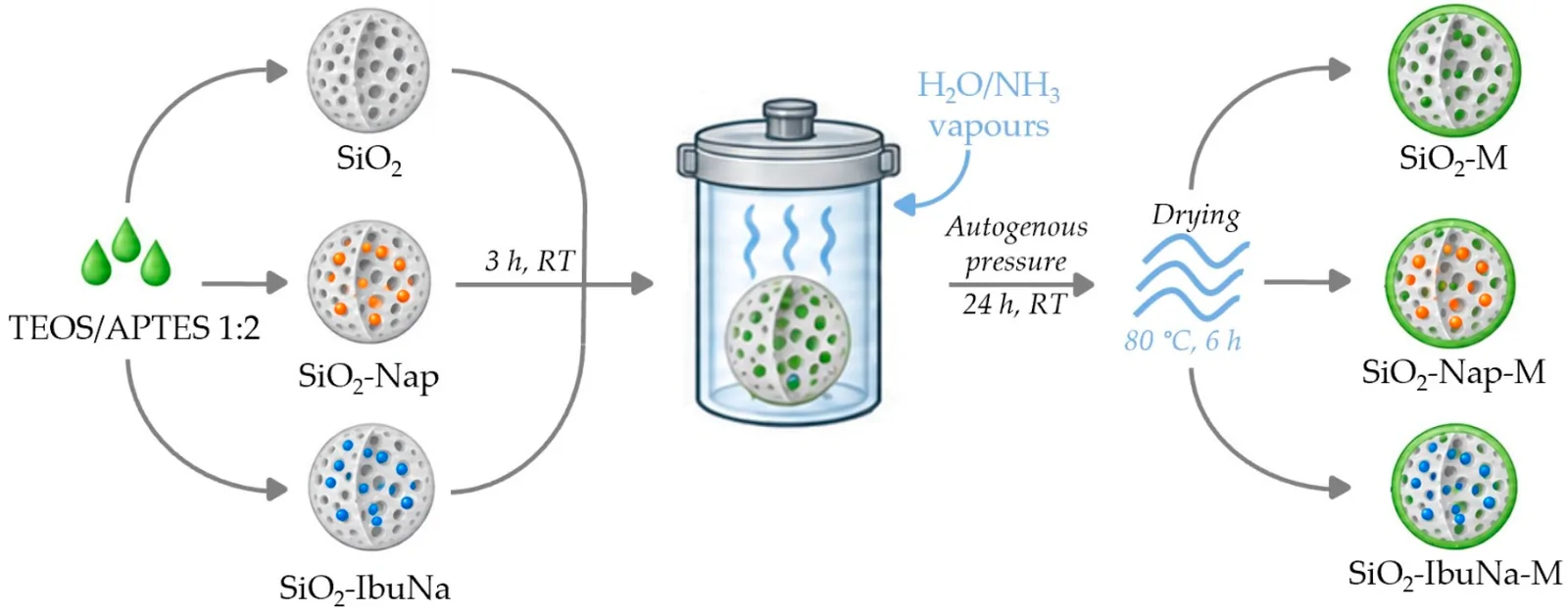
Schematic representation of the synthesis of multicomponent systems via modification of SiO_2_, SiO_2_-Nap, and SiO_2_-IbuNa by introducing hybrid silica gel functionalised with 3-aminopropyl groups.

The SiO_2_, SiO_2_-Nap, and SiO_2_-IbuNa samples were modified by introducing hybrid silica gel functionalised with 3-aminopropyl groups. First, TEOS and APTES were mixed in a 1:2 molar ratio (no longer than one hour before use). This solution was added to the pellets of each sample (i.e., SiO_2_, SiO_2_-Nap, and SiO_2_-IbuNa) in small portions (less than 0.2 mL), and the volume was adjusted so that the samples imbibed thoroughly, with no excess TEOS and APTES solution beyond the pellets. Next, pellets saturated with alkoxysilanes (2 g of alkoxysilanes per 1 g of SiO_2_, 1.6 g per 1 g of SiO_2_-Nap and 1.8 g per 1 g of SiO_2_-IbuNa) were left for 3 h at room temperature in sealed containers and, subsequently, they were separately placed in the closed vessels and subjected to the vapour mixtures of water and ammonia (10 mL 3.25 mol L^−1^ NH_3_(aq) per 1 g of precursors mixture) vapour at autogenous pressure and room temperature for 1 day. Afterwards, the samples were dried at 80 °C for 6 h. The final materials were denoted as SiO_2_-M, SiO_2_-Nap-M, and SiO_2_-IbuNa-M, respectively. Scheme 3 illustrates the synthesis of SiO_2_-M, SiO_2_-Nap-M and SiO_2_-IbuNa-M.

### 2.3. Methods of Analysis

The parameters characterising the porosity of the initial SiO_2_ were determined by nitrogen adsorption/desorption measurements at −196 °C (LN_2_) using the ASAP 2420 analyser (Micromeritics, Norcross, GA, USA). Prior to measurement, the SiO_2_ sample was dried overnight at 200 °C under vacuum. The specific surface area (S_BET_) was established by the BET method [44], the total pore volume (V_p_) was estimated from a single point on the adsorption isotherm at the relative pressure of about 0.99 p/p_0_, and the plot of the corresponding pore size distribution (PSD) was obtained from the desorption branch of the N_2_ isotherms using the BJH model [45].

Positron annihilation lifetime spectroscopy studies were carried out with the use of a digital PALS spectrometer (Maria Curie-Sklodowska University, Lublin, Poland). Two layers of the investigated sample, surrounding a 5 µCi ^22^Na positron source, were placed and degassed inside a custom-designed vacuum chamber equipped with a turbomolecular pump (*p* < 10^−4^ Pa). Photons originating from positron creation inside the source and positron/positronium annihilation inside the sample were collected, respectively, by two scintillation detectors (Scionix, Bunnik, The Netherlands) equipped with Ø1″/1″ BaF_2_ scintillators. Pulses from the detectors were processed with an Agilent U1065A digitiser (Acqiris, Geneva, Switzerland) at a sampling rate of 4 GS s^−1^ and then analysed with dedicated software [46]. A total of 2.2 × 10^7^ was collected in each lifetime spectrum, ensuring high accuracy of further data analysis [47]. The average time resolution of the spectrometer was (198 ± 2) ps. Obtained PAL spectra were analysed with the MELT programme [48], allowing lifetime distributions to be obtained, which were then recalculated into pore size distributions using porosimetry equations [49].

X-ray diffraction (XRD) patterns of the silica carrier, solid dispersions of naproxen and sodium ibuprofen, and the multicomponent systems were obtained using an Empyrean X-ray diffractometer (PANalytical, Malvern, UK) with Cu Kα radiation (λ = 0.154 nm).

Thermal analysis was performed on a STA (Simultaneous Thermal Analysis) 449 F1 Jupiter (Netzsch, Selb, Germany) instrument. The TG-DSC type S sample carrier was used in the study. The heating rate was 10 °C min^−1^. The temperature ranged from 30 °C to 1000 °C. Measurements were carried out separately in both synthetic air and helium atmospheres of purity 6.0 (Air Liquide, Kraków, Poland). After each crucible change—and before the measurement—the furnace chamber was purged of atmospheric gases, filled with the selected gas, and conditioned for 25 min to stabilise the initial conditions. The gas flow rate was 50 mL min^−1^. Before the actual measurements, temperature calibration was performed using In, Sn, Bi, Zn, Al, and Au (Netzsch, 99.9% purity) as temperature standards. Sapphire (Netzsch) was used for sensitivity calibration. The temperature calibration was also verified using In and Al standards, and the measured transition temperatures agreed with the reference values within ±0.5 °C. The average sample mass was (5.01 ± 0.09) mg and (11.88 ± 2.69) mg for pure drugs and for SiO_2_ with drugs, respectively. Although the drug amounts were comparable, the higher total mass may influence heat and mass transfer and hence the measured peak temperatures. Accordingly, thermal stabilisation refers here to delayed thermal degradation under the applied experimental conditions. The empty alumina crucible was used as a reference. The gaseous products emitted during heating were analysed using both a quadrupole mass spectrometer QMS 403 D Aëolos (Netzsch, Selb, Germany) and a Tensor 27 FTIR spectrometer equipped with a TGA–IR interface (Bruker, Karlsruhe, Germany), coupled on-line to the thermal analyser. FTIR spectra were recorded over 650 cm^−1^–4500 cm^−1^ at a resolution of 4 cm^−1^ with 16 scans per spectrum. The FTIR transfer line and gas cell were maintained at 200 °C. The mass spectrometer was coupled online to the thermal analyser via a quartz capillary maintained at 300 °C. Electron ionisation was performed at 70 eV, and mass spectra were acquired in scan mode over the *m*/*z* (*m* is the mass of the molecule and *z* is the charge of the molecule in electron charge units) range of 10–200 amu. The recorded data were processed with the NETZSCH Proteus^®^ software, version 6.1, Nist MS Search 2.0 and OPUS version 7.2.

While preparing this work, the authors used the tool “Grammarly” to check grammar and spelling and the OpenAI tool “ChatGPT 5.6” to support literature searches and the preparation of the Graphical Abstract and Schemes illustrating the individual stages of producing the investigated materials. After using these tools, the authors have carefully reviewed all references, edited the output and take full responsibility for the content of the published article.

## 3. Results and Discussion

### 3.1. Porosity of Samples

**Figure A1:**
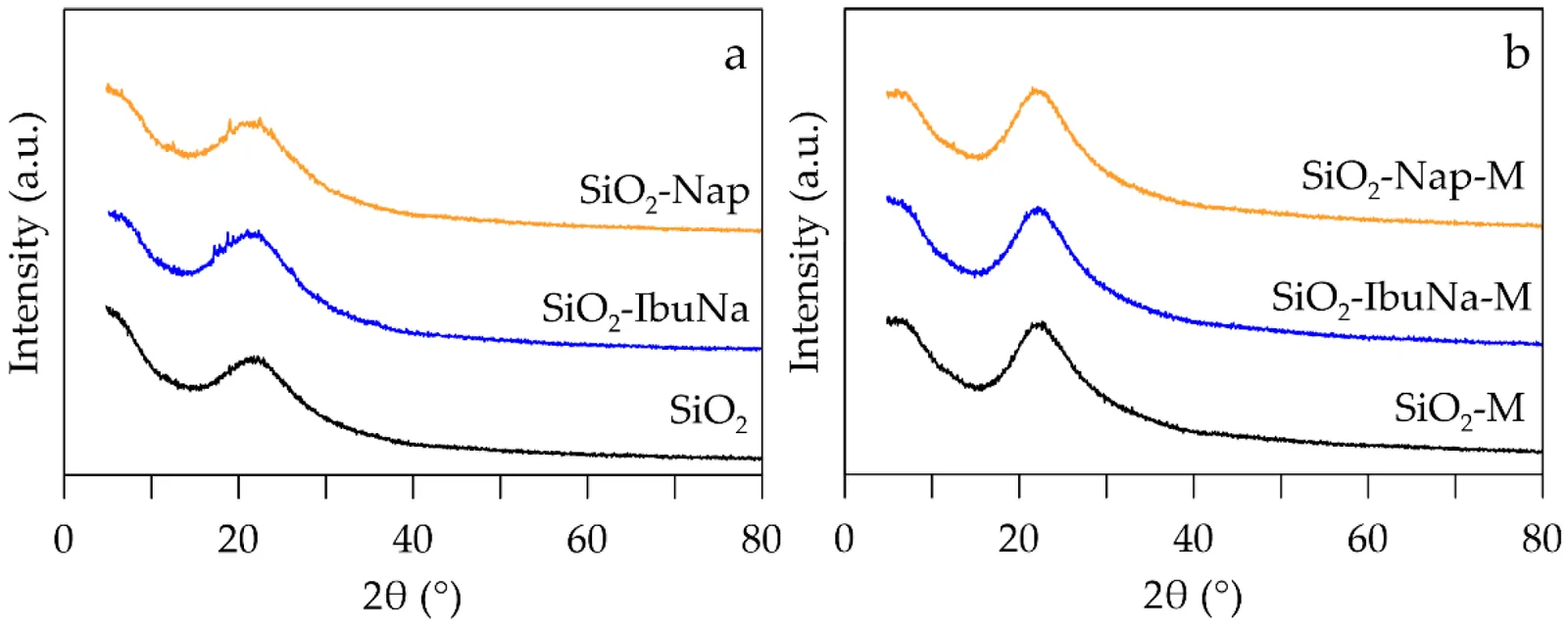
XRD patterns of silica carrier (SiO_2_) and solid dispersions of naproxen (SiO_2_-Nap) and sodium ibuprofen (SiO_2_-IbuNa) (**a**) and the multicomponent systems with drugs, i.e., SiO_2_-Nap-M, SiO_2_-IbuNa-M and drug-free SiO_2_-M (**b**).

**Figure A2:**
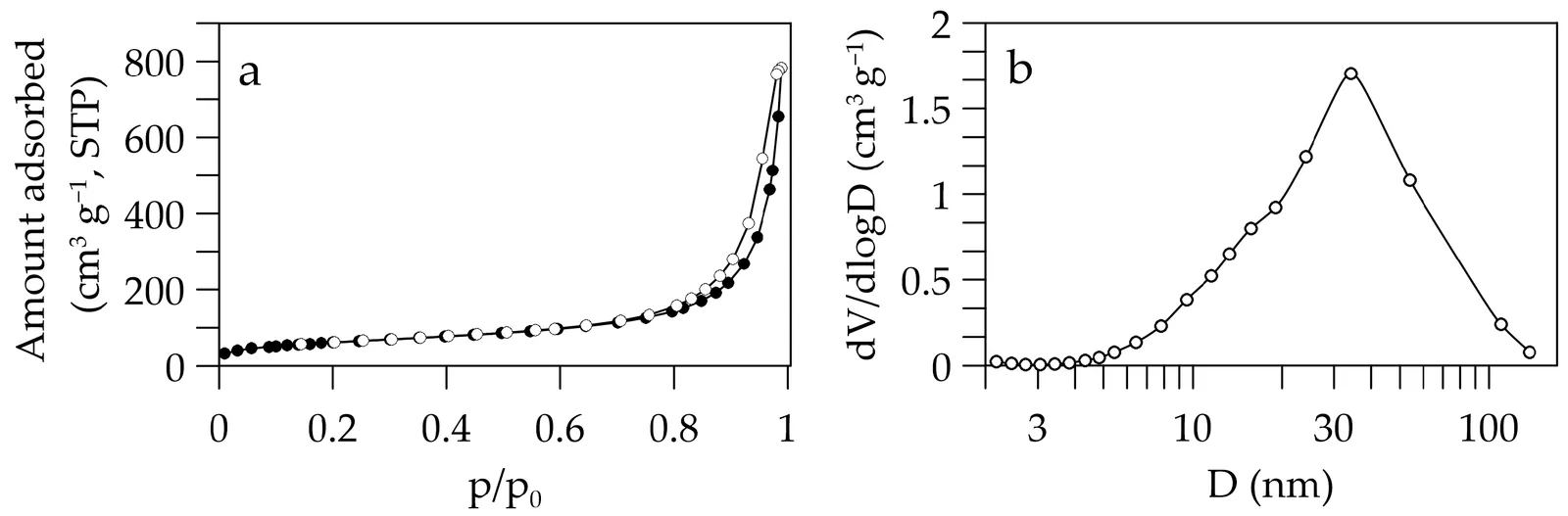
N_2_ adsorption−desorption isotherms (**a**) and the corresponding PSD (**b**) of SiO_2_ pellets.

Using Amberlite^®^XAD7HP beads as a hard template makes it possible to prepare SiO_2_ as spherical pellets [28,50], and their X-ray powder diffraction pattern shows a broad diffuse scattering maximum characteristic of amorphous silica (Figure A1a). As follows from the course of N_2_ adsorption/desorption isotherms presented in Figure A2a, the SiO_2_ pellets are porous, with S_BET_ exceeding 200 m^2^ g^−1^, while V_p_ is 1.21 cm^3^ g^−1^. The pore size distribution of the SiO_2_ pellets shows pores ranging from meso- (peak with a maximum at 34 nm) to macropores (Figure A2b). Therefore, introducing naproxen or sodium ibuprofen from the ethanolic solution into the pores is not a problem. After drug deposition in SiO_2_ and after the NH_2_-SiO_2_ phase formation, the pores of SiO_2_ are filled with these species, which constitute an effective barrier to nitrogen penetration. The free volumes are reduced to such an extent that the LN_2_ measurement becomes inefficient. In this case, PALS results provide more reliable information about the existence of free volumes in SiO_2_, solid dispersions of naproxen and ibuprofen sodium, SiO_2_-Nap-M, and SiO_2_-IbuNa-M samples, as well as about their changes compared with the NH_2_-SiO_2_ phase-free samples.

**Figure 1:**
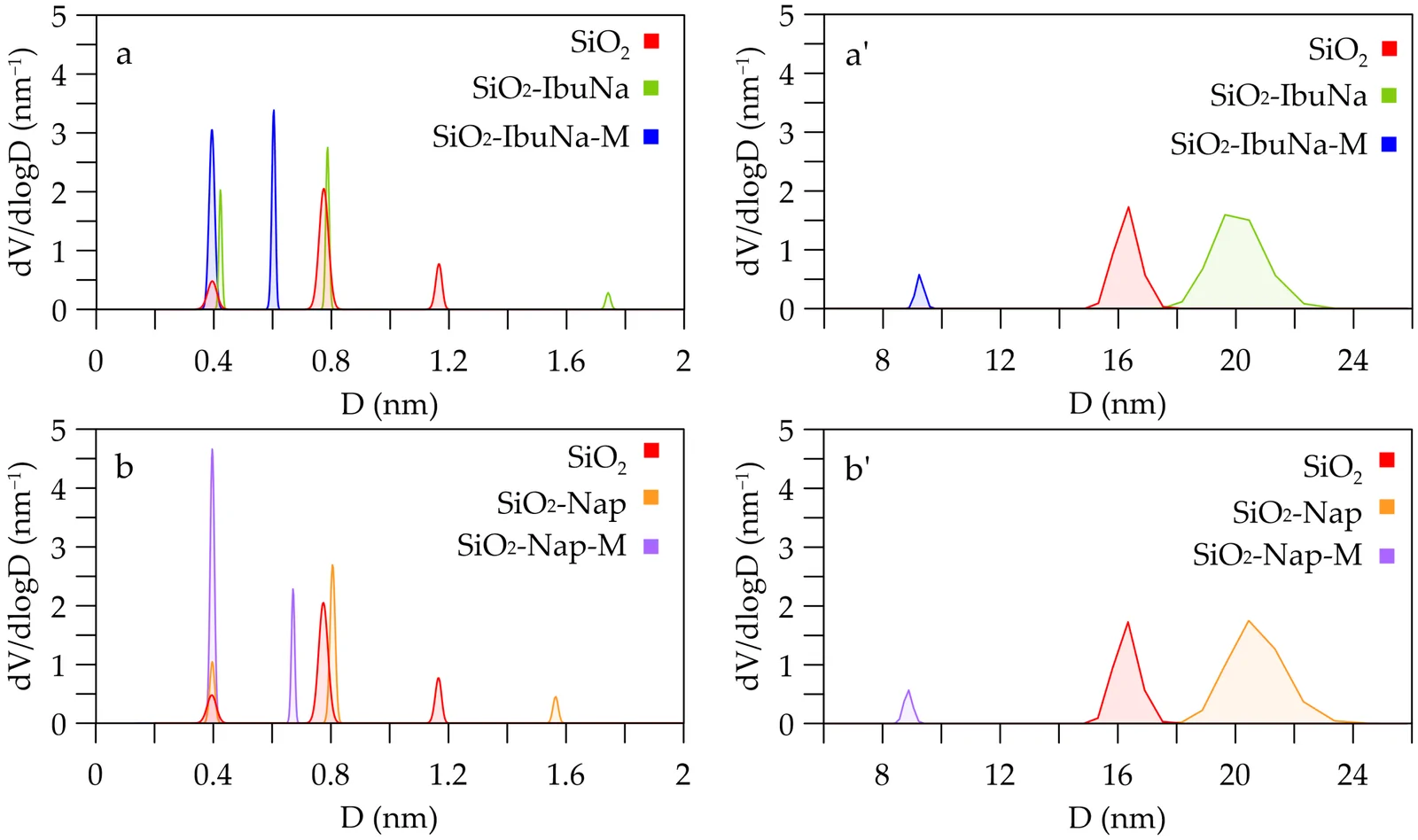
Pore size distributions of nanopores (**a**,**b**) and mesopores (**a’**,**b’**) obtained from positron porosimetry for solid dispersions of ibuprofen sodium (**a**,**a’**) and naproxen (**b**,**b’**).

The pore size distribution for the initial SiO_2_ obtained by positron porosimetry [49] primarily shows a series of three peaks corresponding to subnanometer free volumes (Figure 1a) that are inaccessible to N_2_. These are typical for silica obtained with this method under alkaline conditions [51,52] and indicate a differentiated silica structure. In addition to typical amorphous silica voids between Si and O atoms (approximately 0.4 nm in size), the material contains openwork silica regions with void volumes twice as large and a small admixture of micropores up to three times larger. They originate from the condensation of a silica precursor around the chains of a polymer matrix. Moreover, micropores with an average size of approximately 0.8 nm dominate, with a volume almost equal to that of the mesopores (Table 1). However, comparison with LN_2_ does not indicate the presence of such a large micropore volume. Therefore, they are either closed, or ortho-positronium (o-Ps) annihilating within them is formed in the smaller micropores (0.4 nm) and migrates to the larger ones [53], indicating very thin silica walls.

In the case of mesopores, the PSD obtained from PALS (Figure 1a’,b’) can be compared with that from nitrogen adsorption (Figure A2b). However, the obtained mesopore size distribution is narrower and shifted toward lower diameters. This discrepancy between positron and nitrogen porosimetry results indicates a highly interconnected, branched network of pores of different sizes, in which o-Ps moves freely, traversing regions of varying sizes before annihilation. As a result, we obtain a narrow peak corresponding to the average o-Ps lifetime [54]. The position of its maximum, lower than for nitrogen, is likely due to PALS’s lower sensitivity to macropores, which are not included in the o-Ps annihilation model for the averaged lifetime.

Adding both sodium ibuprofen and naproxen results in a very similar change in porosity. The relative volume of large micropores (0.8 nm and >1 nm) decreases in favour of mesopores. However, the decrease in their volume appears to be slightly greater in the case of IbuNa (Table 1). In turn, the average mesopore size increases noticeably. This indicates that the smaller mesopores are filled with the drug, but the narrow distribution of the mesopore peak in PSD (Figure 1) suggests that the remaining empty mesopore network is still connected, allowing o-Ps to move between pores of different sizes. The loss of volume in the large micropores suggests drug distribution on the silica walls and possibly penetration into subnanometer voids. The XRD patterns of SiO_2_-IbuNa and SiO_2_-Nap (Figure A1a) show weak, poorly resolved diffraction peaks overlapping the broad diffuse scattering maximum characteristic of SiO_2_, which, in turn, prevents reliable determination of their crystalline domain size. These reflections suggest the presence of some crystalline drug, probably the fraction outside the SiO_2_ pores.

The final stage of material preparation, i.e., introducing the amine-functionalised silica phase, results in a drastic reduction in both the size and volume of the mesopores. As expected, the peak corresponding to highly condensed silica increases significantly, confirming its formation in a bulk form. Silica also partially fills the micropores, causing the disappearance of the group of pores larger than 1 nm and shifting the peak from 0.8 nm to 0.6–0.7 nm. Interestingly, silica condensation appears to proceed somewhat differently in the presence of sodium ibuprofen than in the presence of naproxen. In the latter case, fewer but larger micropores seem to remain from the group with an initial size of 0.8 nm. In contrast, after forming the amine-functionalised silica phase, weak diffraction reflections overlapping the broad peak characteristic of amorphous SiO_2_ are no longer resolved in the XRD patterns of both multicomponent systems (Figure A1b). However, the reason for this is difficult to determine.

### 3.2. Thermal Analysis of Investigated Systems

The thermal properties of all solid dispersions of naproxen and sodium ibuprofen in porous silica pellets before and after NH_2_-SiO_2_ phase manufacture from amine-functionalised silica gel in oxidative and non-oxidative atmospheres were assessed through TG/DSC analysis, and simultaneous analysis of FTIR/MS of evolved gases provided complementary information on the thermal events. Thermal analyses of neat naproxen [55,56,57,58,59] and sodium ibuprofen [60,61,62] have already been reported. However, in order to present data for a comprehensive investigation of the thermal stability of both drugs and their solid solutions (studies on sodium ibuprofen are particularly scarce) in helium and synthetic air, over a broad temperature range, with simultaneous detection and analysis of the evolved gases using FTIR and MS spectroscopy, studies are needed. For this reason, and to facilitate discussion and comparison of the studied systems, thermogravimetric analyses of both neat drugs were performed under the same conditions as for the other samples.

#### 3.2.1. Thermal Analysis of Naproxen

**Figure 2:**
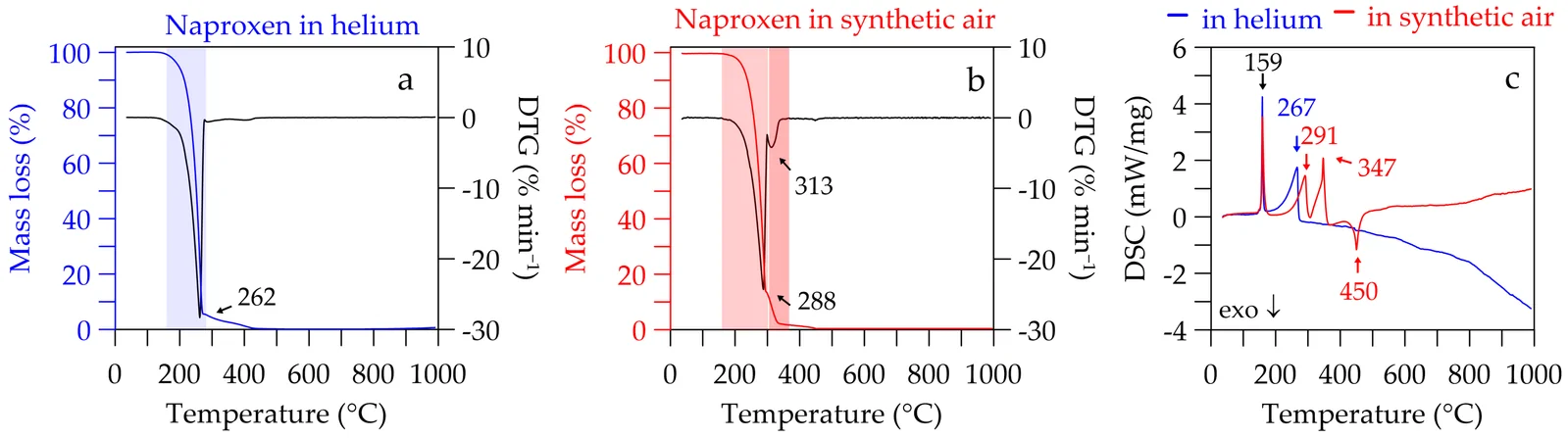
TG-DTG curves of naproxen heated in helium (**a**) and in synthetic air (**b**), and DSC of naproxen measured in both atmospheres (**c**). The blue and red rectangles indicate the temperature ranges discussed below.

The TG/DTG (Figure 2a,b) curves reveal that the measured onset temperatures of naproxen transformation of 156 °C in synthetic air and helium are comparable to those reported elsewhere in nitrogen [55,56,59]. DTG curve shows the main mass loss of naproxen between 155 °C and 276 °C in an inert atmosphere (Figure 2a, blue rectangle), while in synthetic air, after a main mass loss which occurs in the range of (156–297) °C (Figure 2b, light red rectangle), a smaller one is recorded in the range of (297–368) °C (Figure 2b, dark red rectangle). The mass loss of this drug exceeds 99.0% in both gases. DSC measurement (Figure 2c) shows that the degradation of naproxen in helium at all stages is an endothermic process. The first sharp endothermic peak corresponds to the melting of naproxen at 159 °C (peak maximum), while the second one, at about 267 °C, is due to overlapping thermal degradation and volatilisation processes. In turn, under other experimental conditions, additional effects were observed at higher temperatures (above approximately 450 °C) [55,59].

**Figure A3:**
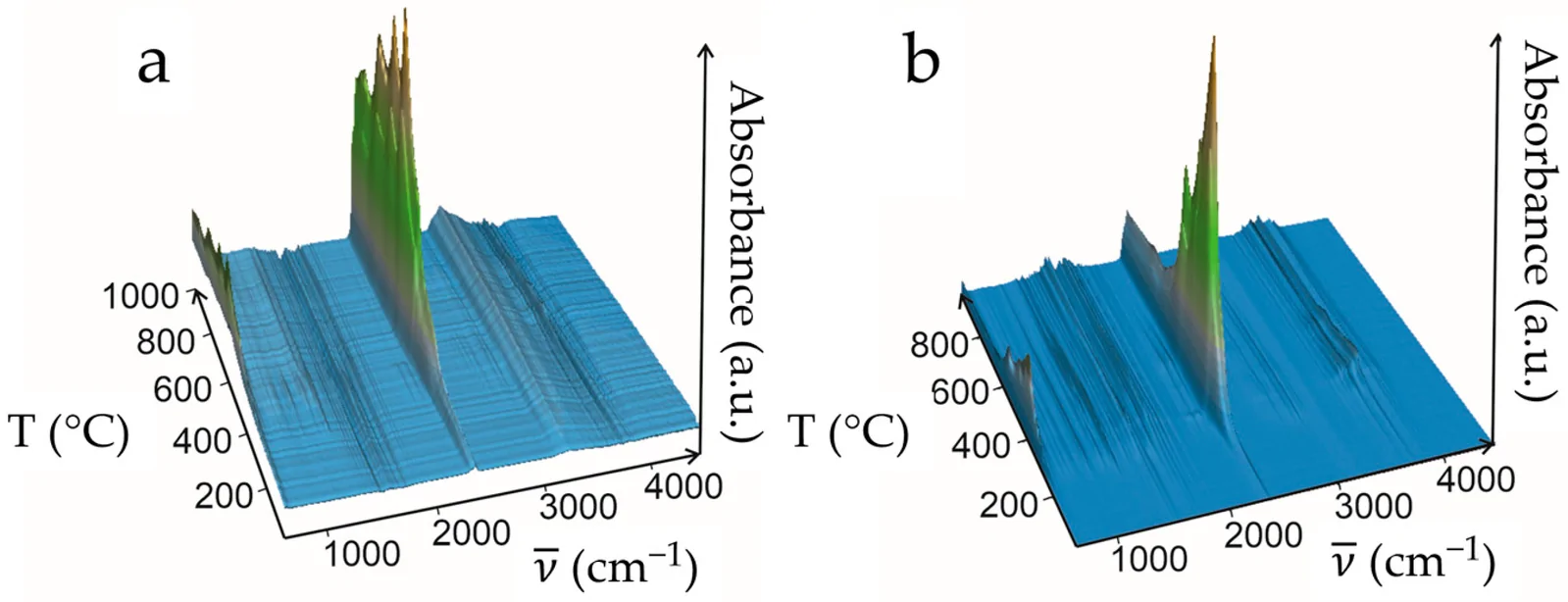
3D diagrams of FTIR spectra of volatiles formed during the heating of naproxen in helium (**a**) and in synthetic air (**b**). The colours represent different absorbance intensities.

Considering the 3D diagram of FTIR spectra (Figure A3a), it can be assumed that the thermal degradation of naproxen in helium generally proceeds towards the formation of high-mass gaseous products, which then defragment at higher temperatures and evaporate rapidly from the sample in the TG/DSC oven. However, they probably condense later in the transfer line leading to the FTIR gas cell, which operates at approximately 200—a temperature lower than that in the TG/DSC oven. Possible retention of condensable organic species may affect their recovery in the FTIR cell; however, the origin of the delayed CO_2_ signal remains unresolved. Therefore, CO_2_ (absorption bands at 2360 cm^−1^ and 2320 cm^−1^), but also H_2_O (absorption bands at 3600 cm^−1^ and 1640 cm^−1^), CO (absorption bands at 2170 cm^−1^ and 2111 cm^−1^), as well as trace amounts of hydrocarbons (absorption bands in the range 3000–2800 cm^−1^) were only detected above 400 °C (Figure A3a) when no further mass loss was observed (Figure 2a). The lack of characteristic bands that could be related to propionic acid or 2-methoxynaphthalene in FTIR (Figure A3a) and which were reported for naproxen heated in dynamic N_2_ [59], suggests that under the TG–FTIR transfer conditions used, helium alters the relative contributions of volatilisation, product removal, and secondary reactions, leading to a different apparent evolved-gas profile from that reported under nitrogen.

In synthetic air, the DSC thermogram suggests consideration of a four-stage degradation profile for naproxen (Figure 2c). The first three well-resolved peaks correspond to the endothermic event, while the fourth peak at 450 °C corresponds to the exothermic event, whereby the peak at 159 °C should be assigned to drug melting (Figure 2c). Above this temperature, in the 3D FTIR emission spectra of the evolved gases, absorption bands at 2111 cm^−1^ and 2170 cm^−1^ of low intensity, 2360 cm^−1^, 2323 cm^−1^, 2313 cm^−1^, and 669 cm^−1^ as well as bands in spectrum region of (3800–3600) cm^−1^ and (1600–1400) cm^−1^ appear, indicating that carbon monoxide, carbon dioxide and water, respectively, are the main gases which evolve from the drug during its degradation in air (Figure A3b). QMID (quasi-multiple ion detection) ion current profiles of gases evolved during thermal decomposition of naproxen confirm that H_2_O and CO_2_ were evolved in both atmospheres (Figure A10, ions with *m*/*z* = 18 and 44, respectively).

#### 3.2.2. Thermal Analysis of Sodium Ibuprofen

**Figure 3:**
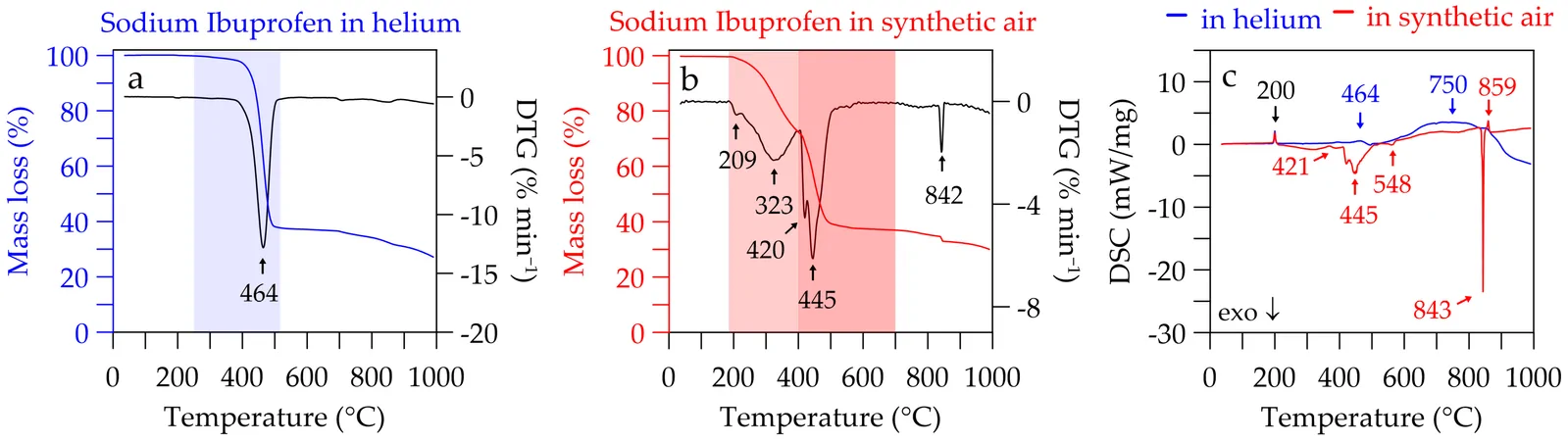
TG-DTG curves of sodium ibuprofen heated in helium (**a**) and in synthetic air (**b**), and DSC of sodium ibuprofen measured in both atmospheres (**c**). The blue and red rectangles indicate the temperature ranges discussed below.

Sodium ibuprofen presents entirely different thermal properties than naproxen. First, even when this salt is exposed to temperatures as high as 1000 °C, a solid residue in quantities exceeding 25% remains at the end of the experiments in both gases (Figure 3a,b). Second, it is much more thermally stable than naproxen under both oxidative and non-oxidative atmospheres.

TG curve in helium reveals that sodium ibuprofen is thermally stable up to 261 °C in principle; only ~1% mass loss is detected, after which mass loss occurs in the range of (261–509) °C (Figure 3a, blue rectangle), corresponding to about 61% of the initial mass (Figure 3a). An additional 10.7% mass loss accompanies further heating of the drug in helium to 1000 °C. Sodium ibuprofen degradation in synthetic air follows a more complex course, starting at approximately 199 °C. The DTG curve in Figure 3b shows two-stage mass loss between 199 °C and 404 °C (Figure 3b, light red rectangle), corresponding to a total of 28% of the starting mass. Additionally, the two-stage mass loss occurs in the (404–700) °C range (Figure 3b, dark red rectangle), as two well-resolved peaks (at 420 °C and 445 °C) are visible on the DTG curve (Figure 3b). Within this range, the total mass loss is 35%. Heating sodium ibuprofen in air above 700 °C is accompanied by further mass loss (about 7%), and a peak with a minimum at 842 °C is present on the DTG curve.

**Figure A4:**
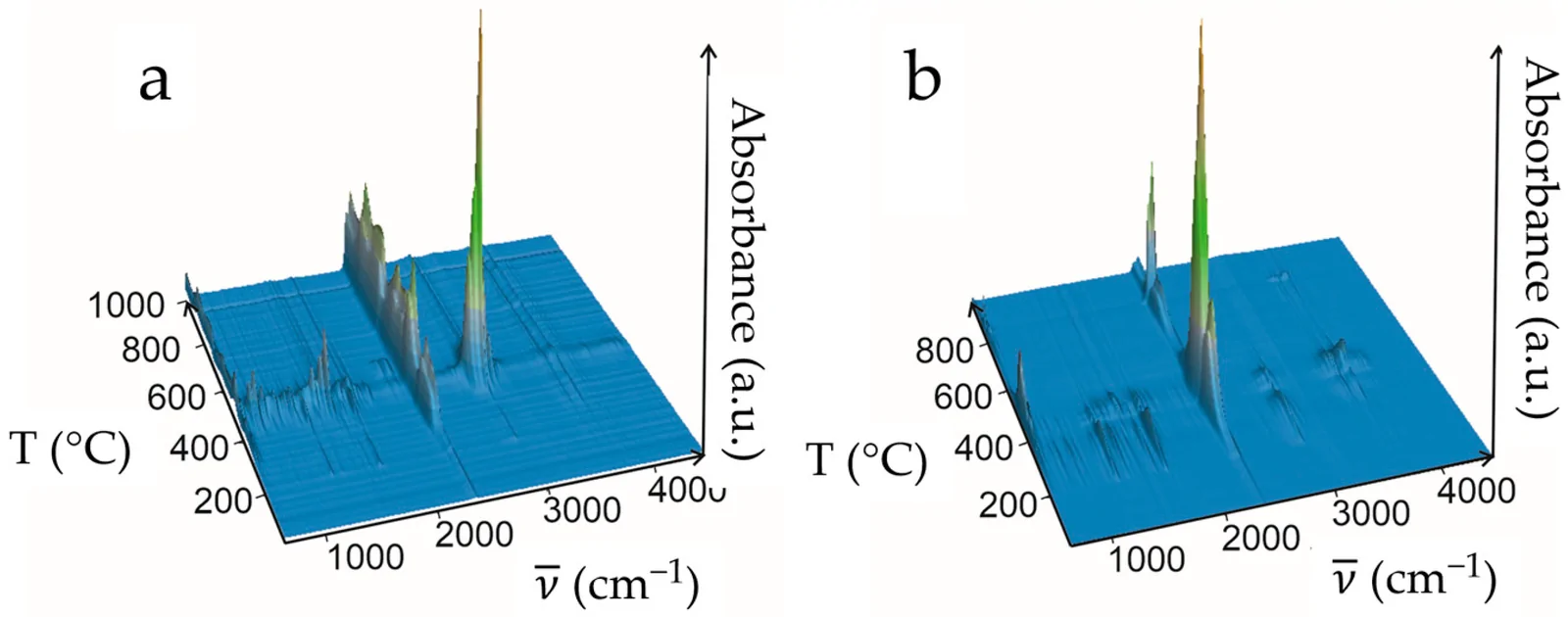
3D diagrams of FTIR spectra of volatiles formed during the heating of sodium ibuprofen in helium (**a**) and in synthetic air (**b**). The colours represent different absorbance intensities.

From the DSC curve (Figure 3c), it is clear that in helium, the thermal decomposition of sodium ibuprofen occurs in an endothermic way. Since the dehydration endothermic hump is invisible below 100 °C, and only a single sharp melting endotherm appears at 200 °C on the DSC, it is reasonable to assume that the tested drug sample is anhydrous racemic (R,S)-(±) sodium ibuprofen with an R:S molar ratio of 1:1 [60,61]. This assumption is confirmed by the 3D diagrams of both the FTIR spectrum (Figure A4a) and the QMID ion current profile (Figure A11a), which indicate that volatiles begin to emit only above 201 °C. Initially, low-intensity signals corresponding to carbon dioxide (i.e., absorption bands at 2360 cm^−1^, 2323 cm^−1^, 2308 cm^−1^, and 669 cm^−1^ in FTIR and the molecular ion of *m*/*z* = 44) and water (molecular ion of *m*/*z* = 18) appear. An increase in temperature above 400 °C results in signals in the QMID profiles and mass spectrum (Figure A11a,b), accompanied by FTIR absorption bands tentatively assigned to 2-(4-isobutylphenyl) propionic acid (i.e., ibuprofen in its acidic form, Figure A4a). The maxima of these signals occur at approximately 464 °C and disappear above 568 °C (Figure A4a and Figure A11a). Only signals corresponding to carbon dioxide and water are detected at the end of the experiment. These results support the interpretation that heating racemic sodium ibuprofen in helium above 400 °C leads to thermal decomposition of the salt and emission of volatile ibuprofen. Surprisingly, the 3D diagram of the FTIR spectra of gases evolved during the heating of sodium ibuprofen in ultra-pure synthetic air presented by Mercurio et al. [62] is very similar to that obtained when heating sodium ibuprofen in helium.

Figure A4b and Figure A11c suggest that the mechanism of racemic sodium ibuprofen degradation under oxidative conditions differs from that under non-oxidative conditions and from the one presented by Mercurio et al. [62]. On the DSC curve (Figure 3c), only two endothermic events at 200 °C and 859 °C are visible, the first of which corresponds to the melting process of sodium ibuprofen. Other events are exothermic, and the one at 843 °C is the most intense. A reasonable explanation for high-temperature events (from 800 °C to 900 °C) for an organic molecule is quite difficult. However, CO_2_ and H_2_O are the only gases that evolved above 800 °C, taking into account the 3D FTIR spectrum (Figure A4b) and QMS profile (Figure A11c,d). Therefore, the exothermic event at 843 °C may be associated with oxidation of carbonaceous residues formed during sodium ibuprofen degradation in air (as suggested by the CO_2_ signal in Figure A12b), while the endothermic peak at 859 °C may correspond to the melting of a sodium-containing phase, possibly sodium carbonate. In contrast, the 3D FTIR spectrum (Figure A4b) and the QMS profiles (Figure A11c,d) contain much weaker signals that may be attributable to ibuprofen, suggesting that a similar pathway may occur to a limited extent in air.

#### 3.2.3. Thermal Analysis of Solid Dispersions of Drugs (SiO_2_-Nap and SiO_2_-IbuNa)

**Figure 4:**
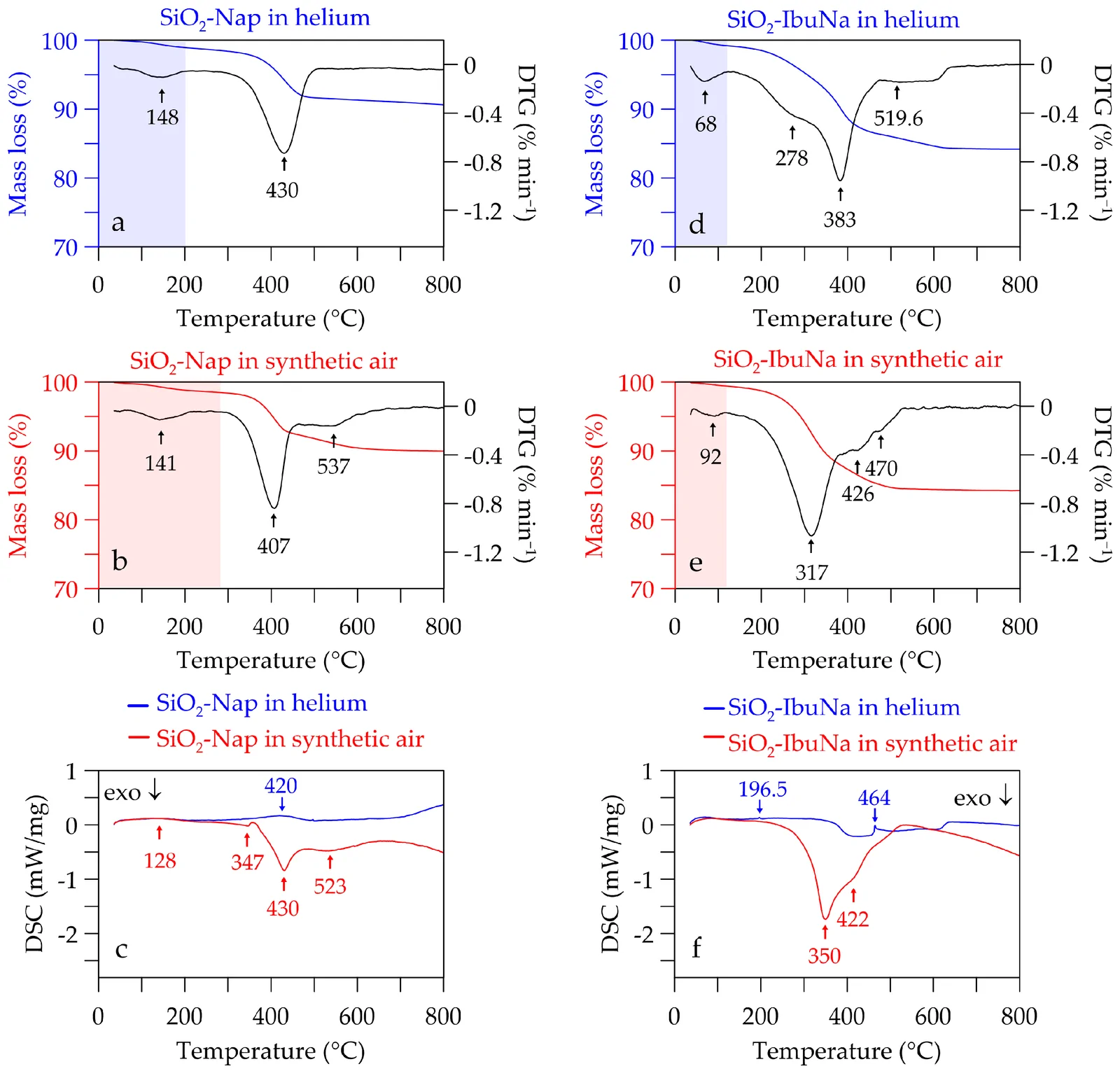
TG-DTG (**a**,**b**) and DSC (**c**) curves of naproxen solid dispersion, and TG-DTG (**d**,**e**) and DSC (**f**) curves of sodium ibuprofen solid dispersion. The blue and red rectangles indicate the temperature ranges discussed below.

SiO_2_ pellets are thermally stable both in synthetic air and helium, as their thermal treatment up to 800 °C results in minimal mass reduction due to water desorption (physically adsorbed as well as formed as a result of dehydroxylation of silica [63,64]). Therefore, SiO_2_ can serve as an inert, thermally stable host, and any differences in the thermal properties of drugs dispersed within SiO_2_ pellets (Figure 4) and bulk drugs (Figure 2 and Figure 3) are solely due to the place and/or way of their immobilisation.

**Figure A5:**
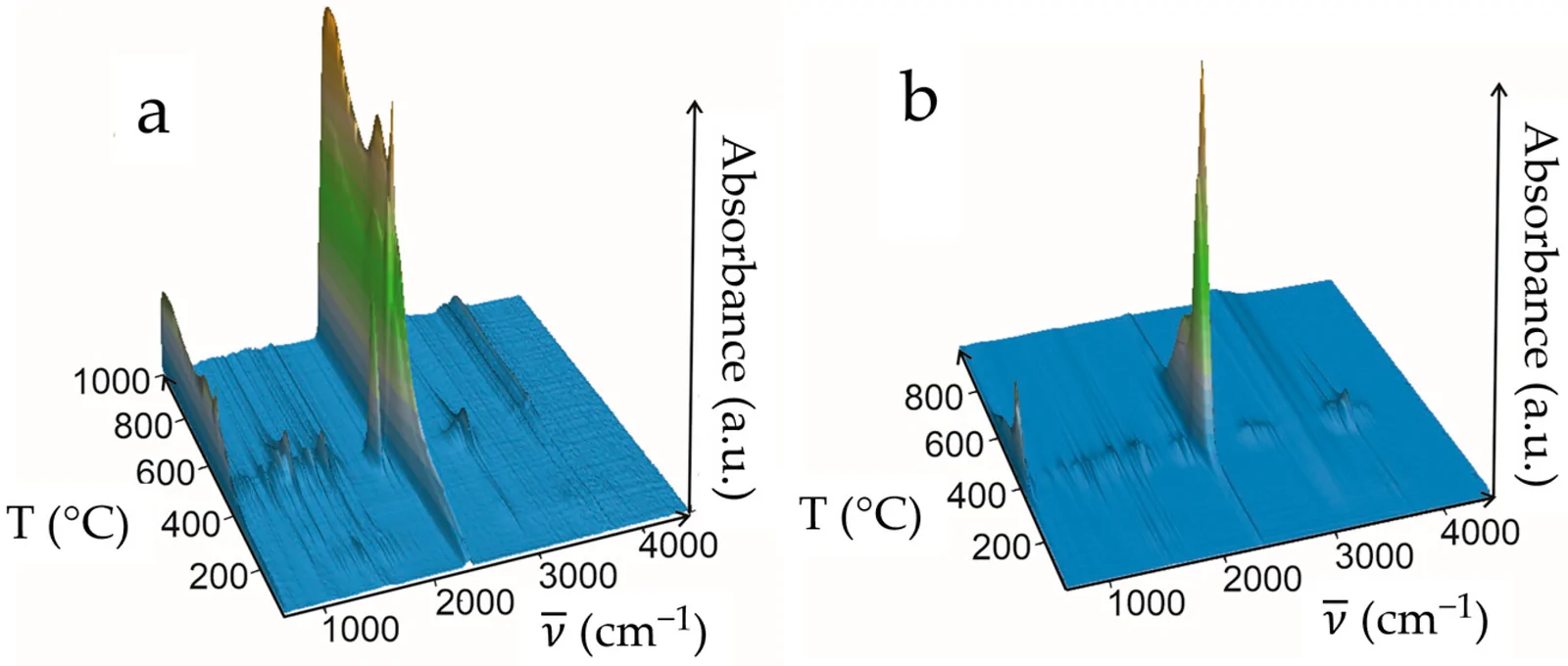
3D diagrams of FTIR spectra of volatiles formed during the heating of SiO_2_-Nap in helium (**a**) and in synthetic air (**b**). The colours represent different absorbance intensities.

The initial mass loss during heating of SiO_2_-Nap and SiO_2_-IbuNa in both gases below 200 °C and 125 °C, respectively (Figure 4a,b,d,e, blue and red rectangles), is negligible, and it is due to water evaporation. The thermograms in the derivative plot recorded for SiO_2_-Nap are similar up to 500 °C, with both peaks occurring at slightly different temperatures (Figure 4a,b). The main mass loss of naproxen immobilised in SiO_2_ takes place at a temperature higher by about 168 °C in He and by about 119 °C in air versus the bulk naproxen (the peak with a maximum at 262 °C in He and 288 °C in air on DTG curves, Figure 2a,b), indicating that silica provides some thermal stabilisation effect, potentially through interactions with naproxen molecules [65,66]. Alternatively, the drug is at least partially confined within the pores of SiO_2_ pellets. As follows from the DSC diagram in He (Figure 4c), SiO_2_-Nap exhibits a three-stage degradation process with only endothermic effects. However, no sharp endothermic peak at 159 °C is observed, which is the naproxen melting point; only a small, very wide peak with a maximum of about 128 °C is visible, which may also suggest confinement of naproxen within silica pores. Cordeiro et al. [65] reported similar effects for naproxen immobilised within the SBA-15 silica host with a wide pore diameter like ours, i.e., broadening of the endotherm and lowering of the melting temperature by about 30 °C. This shift can be attributed to decreased crystal dimensions of the dispersed drug [65,67], as well as to hindered movement and distribution of naproxen molecules due to interaction with the silica walls. The evolved gases include hydrocarbons (in the range of 3000 cm^−1–^2800 cm^−1^) and CO (bands at 2170 cm^−1^ and 2111 cm^−1^), as well as CO_2_ (2360 cm^−1^ and 2320 cm^−1^) (Figure A5a) during decomposition of SiO_2_-Nap above 300 °C in helium. Although naproxen degradation in SiO_2_-Nap in an oxidising atmosphere follows a much more complex course with well-pronounced exothermic events on the DSC curve (Figure 4c), the evolved gases are similar to those recorded in helium, including CO_2_, CO, H_2_O and even hydrocarbons (Figure A5b). They are detected only above 350 °C; above 430 °C, mainly CO_2_ remains in the emitted gases (Figure A5b and Figure A10).

The TG/DTG/DSC profiles of sodium ibuprofen immobilised in SiO_2_ (Figure 4d–f) differ significantly from the TG/DTG/DSC profiles of the neat sodium ibuprofen (Figure 3a–c) in both gases. Firstly, the drug loaded in SiO_2_ is less thermally stable, as mass loss already begins above 125 °C in both gases. Moreover, in a non-oxidising atmosphere, the mass loss of sodium ibuprofen immobilised in SiO_2_ occurs in three stages, with mass losses of approximately 5%, 8%, and 2%, respectively (the DTG curve has maxima at 278 °C, 383 °C, and 520 °C, Figure 4d). In contrast, the bulk sodium ibuprofen decomposes in a single stage (Figure 3a). The small, sharp endothermic peak corresponding to the melting process of sodium ibuprofen in SiO_2_-IbuNa in an inert atmosphere (T_max_ = 196.5 °C, Figure 4f) is shifted marginally to a lower temperature than in the neat drug (T_max_ = 200 °C, Figure 3c). Above 300 °C, the processes are mainly exothermic and low intensity. The exception is a sharp endothermic event at about 464 °C, which is visible, analogous to the neat drug; however, in SiO_2_-IbuNa, this peak is significantly more pronounced. These observations suggest that some of the sodium ibuprofen may be highly dispersed on the outer SiO_2_ surface and/or form tiny clusters with a less organised, less crystalline structure, where the degree of disorder is higher than in neat sodium ibuprofen. As a result, IbuNa in SiO_2_ exhibits different behaviour with increasing temperature than the bulk drug.

**Figure A6:**
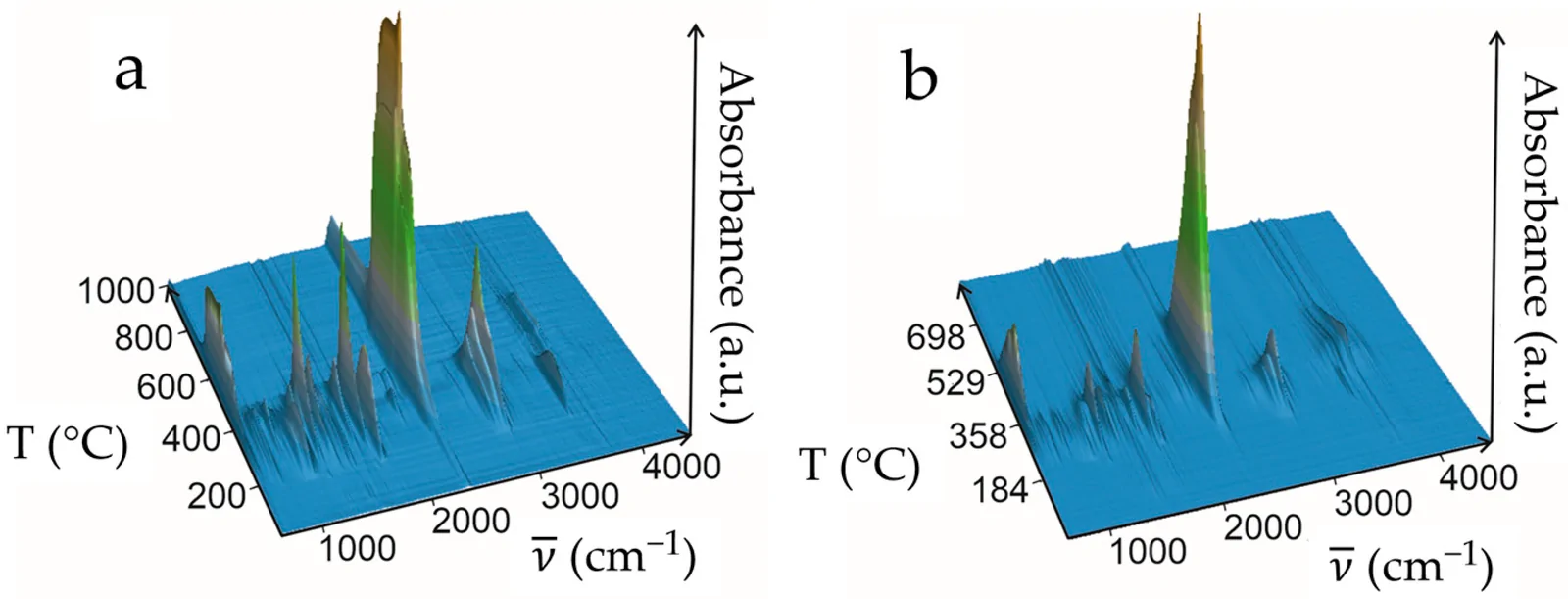
3D diagrams of FTIR spectra of volatiles formed during the heating of SiO_2_-IbuNain in helium (**a**) and in synthetic air (**b**). The colours represent different absorbance intensities.

Analysis of the temperature-dependent 2D/3D plots of QMID (Figure A12 and Figure A13a) and the 3D diagram of the FTIR spectrum (Figure A6a) of evolved gases reveals that many low-intensity signals (i.e., absorption bands in the FTIR spectra and various molecular ions in the MS spectra) appear only above 200 °C. Among these signals, those characteristic of CO_2_ (e.g., absorption bands at 2360 cm^−1^ and 2323 cm^−1^ and 669 cm^−1^ in the FTIR spectra and the molecular ion of *m*/*z* = 44) and water (molecular ion of *m*/*z* = 18) are visible, accompanied by signals that may be attributed to 2-(4-isobutylphenyl) propionic acid (Figure A13b). The intensity of all signals increases with increasing temperature, reaching a maximum at approximately 383 °C, about 100 °C lower than the temperature at which the bulk drug reaches its maximum (Figure A4 and Figure A11). Above ~500 °C, the characteristic signals for ibuprofen in its acidic form disappear, and only signals corresponding to carbon dioxide and water are detected. Therefore, it is likely that in the temperature range from ca. 201 °C to 500 °C, thermal decomposition of dispersed ibuprofen sodium salt on the external SiO_2_ surface occurs, partly to ibuprofen (similar to the case of the bulk drug), which, next, is emitted as a volatile substance, together with water and CO_2_. Water may originate not only from thermal decomposition of the drug, but also from the freshly exposed SiO_2_ surface (previously covered by the sodium ibuprofen molecules). The image of SiO_2_-IbuNa after thermal analysis in helium shows black and white spots on the pellets (Figure A20b). The black spots are likely composed of carbonaceous species; however, their chemical composition is unconfirmed.

DSC of SiO_2_-IbuNa in synthetic air (Figure 4f) exhibits an entirely different course from neat sodium ibuprofen (Figure 3c). First, it lacks endothermic peaks corresponding to the melting of sodium ibuprofen and sodium compounds, respectively. Moreover, only a broad and well-marked exothermic peak with a maximum of about 350 °C, with a shoulder shifted toward a higher temperature (poorly resolved peak with a maximum of about 422 °C), dominates the DSC of SiO_2_-IbuNa. The temperature-dependent 2D plots of QMID (Figure A12 and Figure A13c) and the 3D diagram of the FTIR spectra (Figure A6b) of the gases evolved during the thermal decomposition of SiO_2_-IbuNa show CO_2_ evolution above 260 °C, accompanied by bands in the ranges of (3000–2800) cm^−1^) and (1670–1780) cm^−1^), attributable to hydrocarbons and molecules containing carbonyl groups respectively, possibly volatile ibuprofen (Figure A6b and Figure A13d), similar to the case of SiO_2_-IbuNa heating in helium. All signals cease above ~420 °C, except for the CO_2_ signal.

#### 3.2.4. Thermal Analysis of Multicomponent Systems—SiO_2_-M

**Figure 5:**
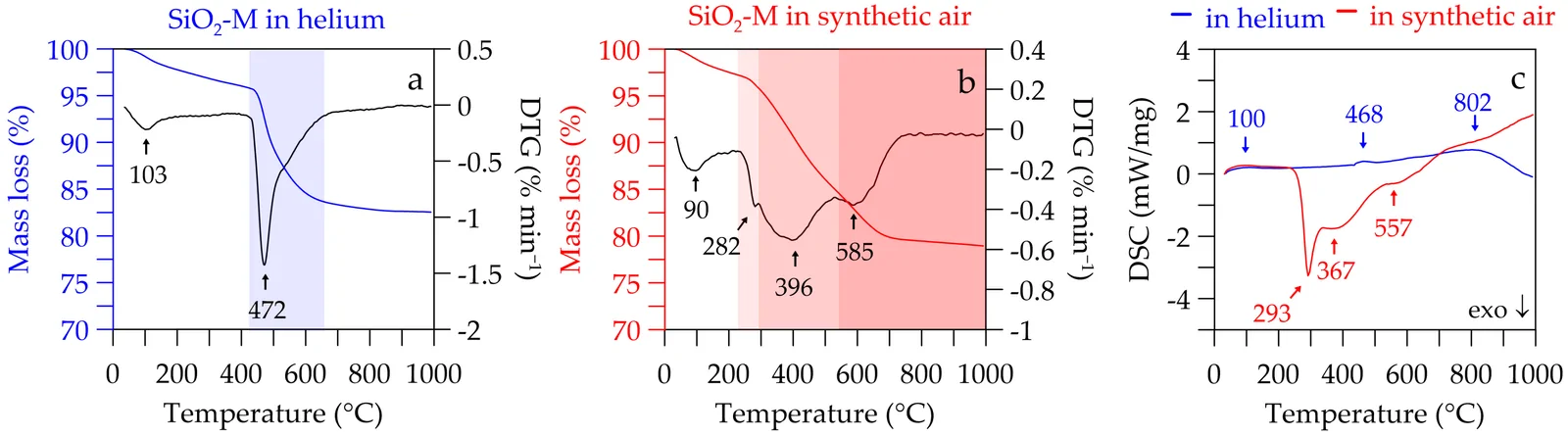
TG-DTG (**a**,**b**) and DSC (**c**) curves of SiO_2_-M heated in helium and air. The blue and red rectangles indicate the temperature ranges discussed below.

Considerable changes in the thermal properties of the solid dispersions SiO_2_-Nap and SiO_2_-IbuNa are observed after manufacturing the NH_2_-SiO_2_ phase composed of the amine-functionalized silica gel. However, to provide a reliable discussion of the results for SiO_2_-Nap-M and SiO_2_-IbuNa-M, the thermal degradation of drug-free SiO_2_ pellets modified with the hybrid silica gel functionalized with 3-aminopropyl groups (i.e., SiO_2_-M) was investigated (Figure 5).

Although a weight loss of less than 3% from room temperature to about 200 °C is observed in helium and synthetic air atmospheres, and thermal treatment up to 1000 °C is accompanied by similar mass loss (i.e., by a further 15% and 19%, respectively), it is evident that the TG/DTG profiles of SiO_2_-M (Figure 5a,b, respectively) have a very different course in both gases. SiO_2_-M shows a single mass loss between 420 °C and 676 °C in an inert atmosphere (Figure 5a, blue rectangle), while in synthetic air after the slight mass loss (less than 1.5%) which occurs in the range of (235–300) °C (Figure 5b, light red rectangle), the significant mass losses, i.e., 11% and 6% are recorded in the ranges of (300–531) °C (Figure 5b, medium red rectangle) and (531–1000) °C (Figure 5b, dark red rectangle), respectively. SiO_2_-M, prepared by treating SiO_2_ impregnated with TEOS and APTES with the vapour mixtures of water and ammonia, exhibits comparable thermal stability in an inert atmosphere to other amine-functionalized silica materials [68,69,70].

**Figure A7:**
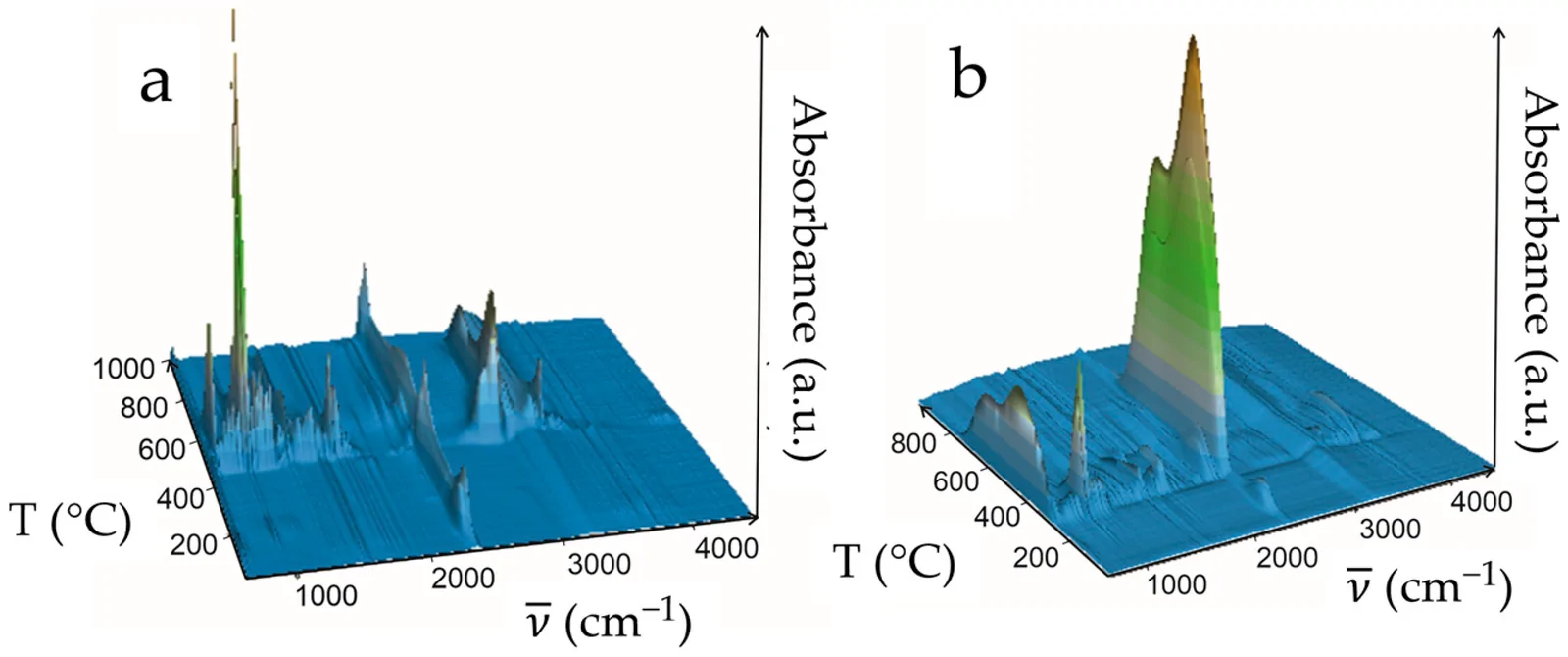
3D diagrams of FTIR spectra of volatiles formed during the heating of SiO_2_-M in helium (**a**) and in synthetic air (**b**). The colours represent different absorbance intensities.

From the DSC curve in helium (Figure 5c), it follows that SiO_2_-M exhibits a three-stage decomposition process with only endothermic effects. The first one, a very weak and broad peak centred at about 100 °C, can be assigned to the desorption of water, ammonia, CO_2_ and CO since, from room temperature up to about 280 °C, molecular ions of *m*/*z* = 15, 16, 17, 18, and 44 are observed in the effluent gas (Figure A10 and Figure A14a) and characteristic bands are observed in FTIR (Figure A7a). The presence of adsorbed CO_2_ is evident, as aminopropyl-functionalised silica gel readily adsorbs CO_2_ [68,71,72,73,74] from ambient air. The presence of H_2_O and NH_3_ results from the synthetic method employed, which uses H_2_O and NH_3_ vapours as triggers for TEOS and APTES hydrolysis and condensation. The second endothermic event recorded in the range of (400–500) °C (Figure 5c) corresponds to the desorption of decomposition products of aminopropyl groups covalently attached to the silica. First, signals of *m*/*z* = 15, 16 and 17 appear in the effluent gas at about 400 °C with low intensity; they intensify at about 463 °C and reach a maximum height at 472 °C (Figure A7a and Figure A10), where the mass loss rate is greatest (minimum on the DTG curve). However, they are visible in the mass spectrum of gases evolved from SiO_2_-M collected only at 477 °C (the shift is related to the *m*/*z* scanning speed) (Figure A15a). These signals suggest early degradation involving amino groups and NH_3_ evolution, possibly through C-N bond cleavage. NH_3_ evolution occurs at temperatures ranging from about 400 °C to 900 °C and is reflected by characteristic bands in the range of (3400–3200) cm^−1^ and peaks at ~930 cm^−1^, ~965 cm^−1^, ~1625 cm^−1^ (Figure A7) [75]. Species with a mass-to-charge ratio of 25, 26, 27, 37, 39, 41, 42, 43, 56, 57, and 79 appear only at about 450 °C on temperature-dependent plots of QMID (Figure A14a and Figure A15a) and disappear at about 680 °C. They may originate from the detachment and decomposition of the hydrocarbon backbone of the silane remaining after -NH group detachment. A similar trend in the thermal decomposition of amine-grafted SBA-15 in air was reported by Khatri et al. [76]. Based on the mass spectrum, it is most likely that the hydrocarbon decomposition occurs through the emission of various low-molar-mass products, but their unambiguous identification is impossible. The third wide endothermic event, with a peak at approximately 802 °C (Figure 5c), may correspond to silica densification, probably the silica introduced during the NH_2_-SiO_2_ phase manufacture. The insignificant change in the TGA profile and the molecular ion at *m*/*z* = 18 in the QMID profiles (Figure A10c and Figure A14a) indicate that water desorption is a result of increasing silica condensation.

The first, low-intensity, endothermic signal on the DSC curve of SiO_2_-M in synthetic air, with a maximum at approximately 100 °C, can be attributed to the desorption of CO_2_ (from ambient air), H_2_O, and NH_3_ (residual vapours used for synthesis). All other events during heating of SiO_2_-M in synthetic air above 200 °C occur only with exothermic effects (Figure 5c). The first event is the most pronounced, and a well-separated peak at about 293 °C is visible in the DSC. The second event appears between 340 °C and 531 °C, and the third one between 531 °C and 700 °C. The QMID profiles (Figure A14b) suggest that heating above 286 °C initiates decomposition of surface NH_2_ groups, with NH_3_ represented by *m*/*z* = 17. The additional ions at *m*/*z* = 26 and 30 (Figure A15b) may originate from low-molar-mass hydrocarbon- and/or amine-containing species [70]. Hence, species such as ethane, acetylene, propane or even ethylenediamine, N-propyl-ethylenediamine, and propylamine cannot be excluded, although their unambiguous identification is impossible. The maximum of the peak corresponding to NH_3_ in the MS spectrum occurs at ca. 325 °C, where the oxidation of hydrocarbons begins, and CO and CO_2_ are detected in reasonable amounts (Figure A7b and Figure A14b). Above 440 °C, NH_3_ is no longer detectable in the effluent gas, but the maximum of the peak corresponding to CO_2_ in the MS spectrum is observed (Figure A14b). Hence, this may indicate that large-scale oxidation of the hydrocarbon backbone of the silane begins only after the -NH groups decompose and continues up to 710 °C (Figure A14b). The study using APTES on SBA-15 medium, conducted by Khatri et al. [76] and other research groups [70,77], leads to similar conclusions.

#### 3.2.5. Thermal Analysis of Multicomponent Systems—SiO_2_-Nap-M and SiO_2_-IbuNa-M—in Helium

**Figure 6:**
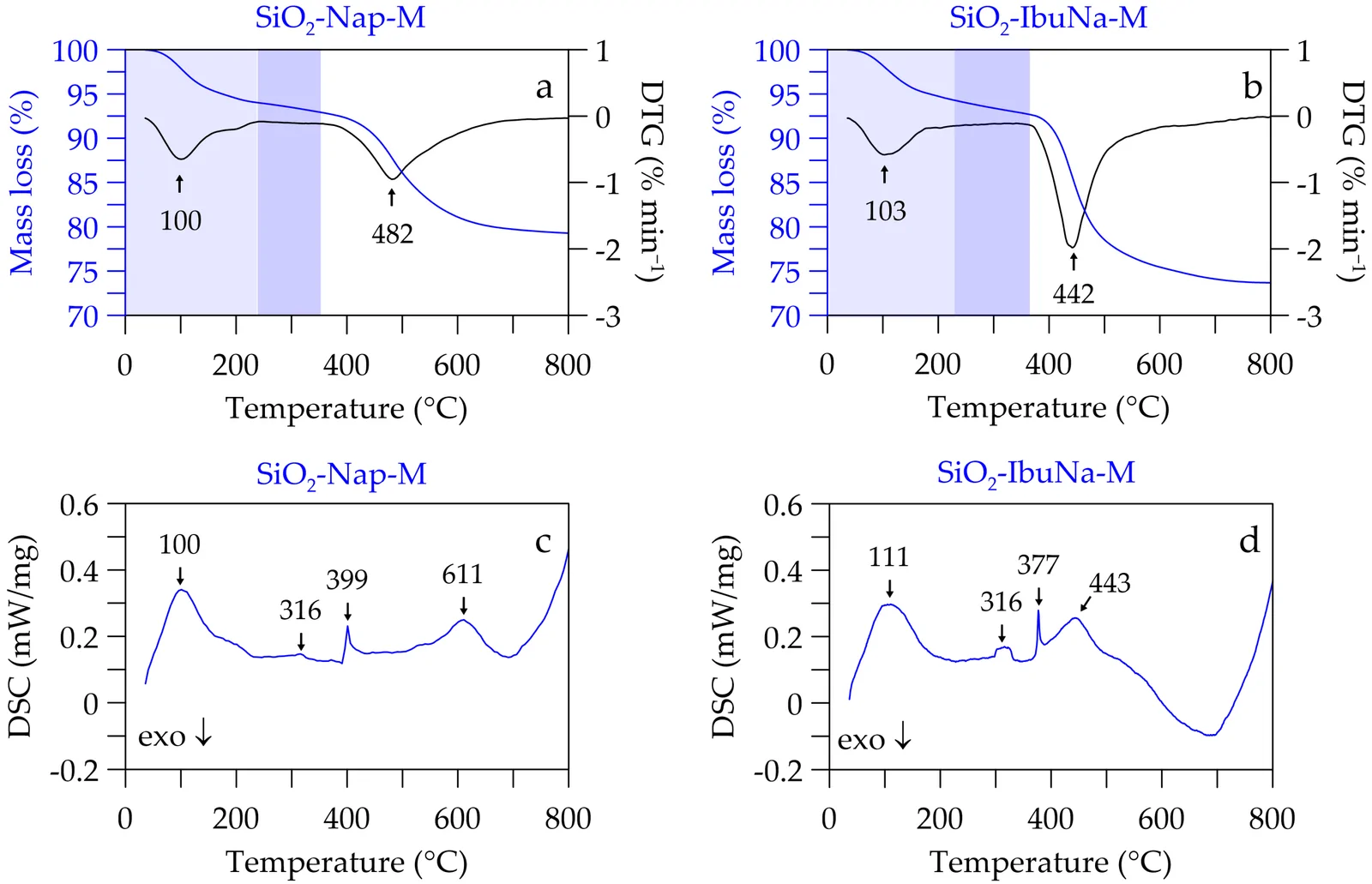
TG-DTG (**a**,**b**) and DSC (**c**,**d**) curves of SiO_2_-Nap-M and SiO_2_-IbuNa-M heated in helium. The blue rectangles indicate the temperature ranges discussed below.

The TG/DTG profiles of SiO_2_-Nap-M and SiO_2_-IbuNa-M recorded in helium and presented in Figure 6a,b, respectively, are similar and resemble the TG/DTG profile of SiO_2_-M (Figure 5a,b). The initial weight loss of SiO_2_-Nap-M and SiO_2_-IbuNa-M occurs when the samples are heated to 230 °C (Figure 6a,b, light blue rectangle) and does not exceed 6%. Further heating to 360 °C (Figure 6a,b, dark blue rectangle) leads to a mass loss of less than 1.5% for both samples. The main mass loss of the SiO_2_-Nap-M and SiO_2_-IbuNa-M samples is detected above 360 °C, and the peaks with maxima at 482 °C and 442 °C, respectively, are visible on the DTG curves. Comparison of the thermograms in the derivative plot recorded for SiO_2_-Nap, SiO_2_-IbuNa (Figure 4), and SiO_2_-Nap-M and SiO_2_-IbuNa-M (Figure 6a,b) indicates that the formation of the amine-functionalized silica gel phase in both solid drug dispersions significantly increases the thermal stability of the multicomponent systems.

DSC profiles of the SiO_2_-Nap-M and SiO_2_-IbuNa-M samples in helium are relatively similar (Figure 6c,d). In both cases, the endothermic peak dominates, with a maximum at about 100 °C (in the case of SiO_2_-Nap-M) and 111 °C (in the case of SiO_2_-IbuNa-M). This corresponds to the desorption of NH_3_, H_2_O and CO_2_, as indicated by peaks in the mass spectrum at a mass-to-charge ratio *m*/*z* = 15, 16, 17, 18 and 44, respectively (Figure A17). These gases, similar to SiO_2_-M, result from the synthesis method used. At a temperature just above 200 °C, following the initial low-temperature event, a poorly resolved endothermic peak is visible in the DSC (with a maximum at about 316 °C in SiO_2_-Nap-M and SiO_2_-IbuNa-M, Figure 6c,d). This peak likely corresponds to a weak effect related to decomposition of both systems, with desorption of small amounts of gases including H_2_O, CO_2_, and NH_3_ (Figure A10 and Figure A12). The primary source of ammonia is the decomposition of the aminopropyl groups. Therefore, the decomposition process of the amine-functionalized silica gel phase in the SiO_2_-Nap and SiO_2_-IbuNa solid dispersions also begins at the surface -NH groups, as in SiO_2_-M; however, it occurs at a much lower temperature. The presence of drugs immobilised in the SiO_2_ pellets may be a contributing factor. Since the decomposition process is similar for SiO_2_-Nap-M and SiO_2_-IbuNa-M—regardless of the drug type present—it can be concluded that the manufacturing process of the phase made of amine-functionalized silica gel proceeded in a similar manner. Furthermore, the attachment and orientation of this NH_2_-SiO_2_ phase on both solid dispersions appear to be similar, despite significant differences in the quantity of naproxen and sodium ibuprofen immobilised.

**Figure A8:**
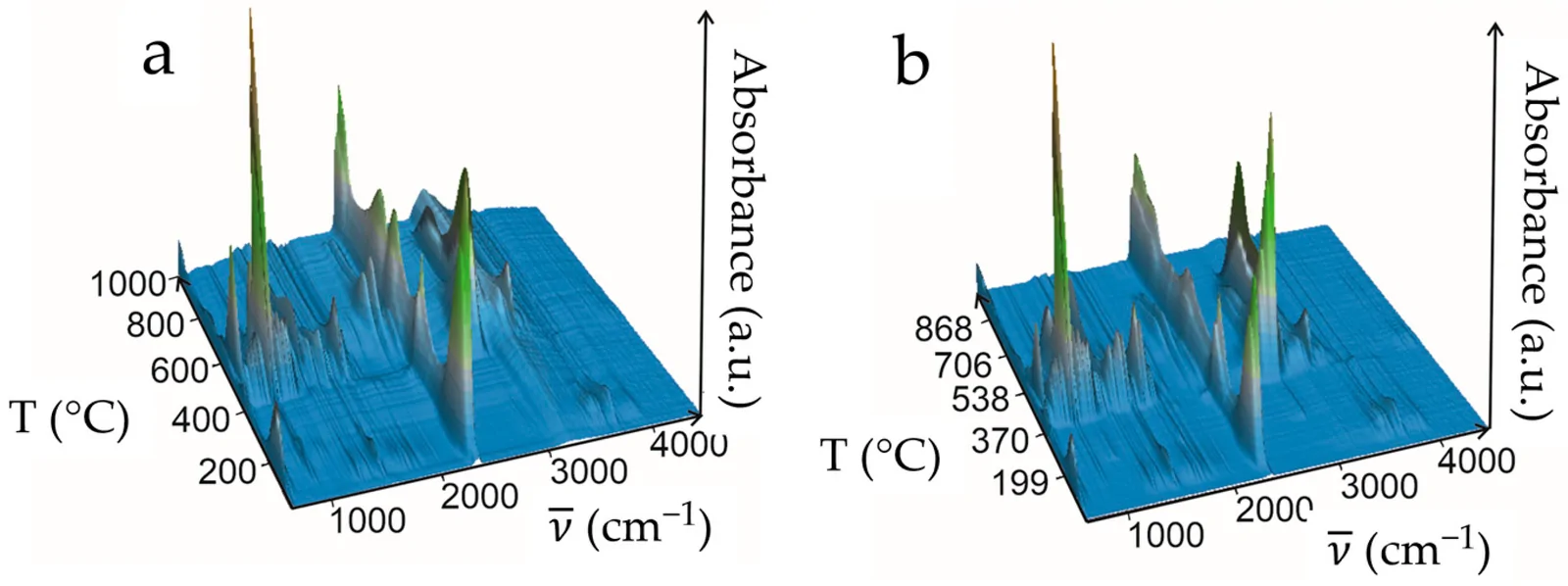
3D diagrams of FTIR spectra of volatiles formed during the heating of SiO_2_-Nap-M (**a**) and SiO_2_-IbuNa-M (**b**) in helium. The colours represent different absorbance intensities.

What distinguishes the DSC of SiO_2_-Nap-M and SiO_2_-IbuNa-M is the presence of the sharp, low-intensity peaks with maxima at 399 °C and 377 °C, respectively, which, in turn, are absent in the DSC of SiO_2_-M. These peaks are followed by endothermic events with a maximum at about 611 °C in the DSC of SiO_2_-Nap-M and about 443 °C in the DSC of SiO_2_-IbuNa-M (Figure 6c,d). It is important to note that endothermic events are observed at a temperature above 400 °C for SiO_2_-M (peak maximum at 468 °C), as well as for the SiO_2_-Nap and SiO_2_-IbuNa solid dispersions (peaks with maxima at 420 °C and 464 °C, respectively). The temperature-dependent 2D plots of QMID ion current curves (Figure A16a) and 3D diagrams of FTIR spectra (Figure A8a) of volatiles formed during the thermal decomposition of SiO_2_-Nap-M indicate that NH_3_ (being the product of the decomposition of the surface -NH from the aminopropyl group) is emitted at temperatures from about 400 °C to 900 °C, similar to SiO_2_-M. It is accompanied by the emission of other gases, including CO_2_, CO, H_2_O and even hydrocarbons (bands in the range of 3000 cm^−1–^2800 cm^−1^) and amines (bands above 3000 cm^−1^) (Figure A8a). Decomposition of the hydrocarbon backbone of the silane remaining after detachment of -NH groups coincides with naproxen decomposition. These processes intensify above 500 °C (Figure A8a, Figure A16a and Figure A18a) and essentially end at about 640 °C. It is not possible to isolate the decomposition processes of naproxen and the 3-aminopropyl groups of the NH_2_-SiO_2_ phase, but it is evident that the decomposition of the organic part of SiO_2_-Nap-M takes place at temperatures higher than those recorded for the neat naproxen and SiO_2_-Nap, and even for SiO_2_-M. The representative image of SiO_2_-Nap-M after thermal analysis in helium (Figure A21a) shows a different appearance from SiO_2_-Nap (Figure A20a) and resembles SiO_2_-M (Figure A20c). The dark residue remaining after heating to 1000 °C may contain carbonaceous products derived from the organic components, which were both naproxen and the 3-aminopropyl groups in the NH_2_-SiO_2_ phase.

The decomposition of SiO_2_-IbuNa-M occurs in a slightly different way (Figure 6d). First, volatiles formed during the thermal decomposition of SiO_2_-IbuNa-M appear just above 400 °C (so at a somewhat lower temperature compared to SiO_2_-Nap-M, Figure A16b). Species with *m*/*z* values belonging to volatile substances resulting from the thermal decomposition of 3-aminopropyl groups, including NH_3_, were similar to those observed in SiO_2_-M (Figure A7a and Figure A15a). However, in addition to the above-mentioned molecular ions, species with *m*/*z* = 105, 115, and 117 were recorded at 444 °C (Figure A18b). Therefore, it is likely that despite the manufacture of the phase composed of the amine-functionalized silica gel in the sodium ibuprofen solid dispersion within SiO_2_, the decomposition process of SiO_2_-IbuNa-M in helium proceeds similarly to the decomposition process of SiO_2_-IbuNa, as suggested by the detection of signals assigned to volatile ibuprofen. From the model of hydrolysis of APTES under basic conditions presented by Chen et al. [78] and Golub et al. [79], it follows that the primary particles originating from TEOS and APTES have a complex structure and tend to agglomerate into larger systems. Most probably, molecules of sodium ibuprofen in SiO_2_-IbuNa, probably mainly those which were not immobilised in SiO_2_ pores, may instead have been trapped in the hybrid NH_2_-SiO_2_ phase during the formation of SiO_2_-IbuNa-M. It is likely that during heating of SiO_2_-IbuNa-M in helium, they are desorbed in the form of ibuprofen. Unlike the SiO_2_-Nap-M sample, the representative image of SiO_2_-IbuNa-M after thermal analysis in helium (Figure A21b) resembles the image of SiO_2_-IbuNa. This indicates that after high-temperature treatment of SiO_2_-IbuNa-M in helium, the SiO_2_ pellets are covered only partially with carbonaceous species, which, in turn, are formed after decomposition of ibuprofen and the 3-aminopropyl groups in the NH_2_-SiO_2_ phase.

#### 3.2.6. Thermal Analysis of Multicomponent Systems—SiO_2_-Nap-M and SiO_2_-IbuNa-M—in Synthetic Air

**Figure 7:**
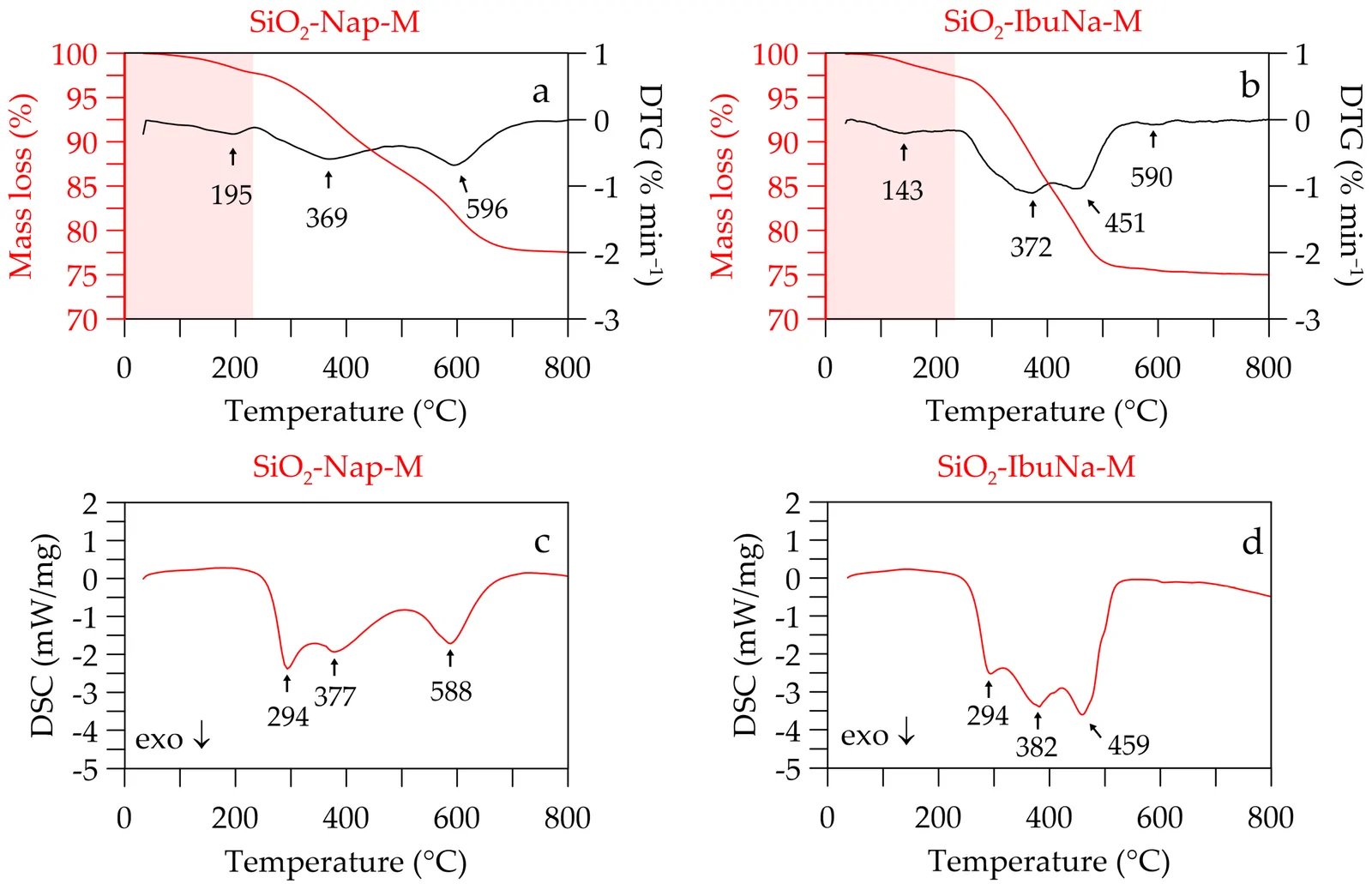
TG-DTG (**a**,**b**) and DSC (**c**,**d**) curves of SiO_2_-Nap-M and SiO_2_-IbuNa-M heated in synthetic air. The red rectangles indicate the temperature ranges discussed below.

The mass reduction in SiO_2_-Nap-M and SiO_2_-IbuNa-M occurs in three stages under oxidative conditions since three well-resolved peaks are visible on the DTG curves of both samples (Figure 7a,b). The course of these DTGs somewhat resembles that of DTG for SiO_2_-M, whereby the position of maxima on the DTG is slightly shifted in reference to SiO_2_-M. In contrast, they differ significantly from the TG/DTG recorded in an inert atmosphere. The initial mass loss, less than 3%, occurs up to approximately 240 °C for both samples (Figure 7a,b, red rectangle). The peaks with maxima at 195 °C and 143 °C are present on the DTG curves for SiO_2_-Nap-M and SiO_2_-IbuNa-M, respectively. They are significantly shifted in comparison with the SiO_2_-M sample. Significant mass loss of SiO_2_-Nap-M occurs when the sample is heated to ca. 500 °C, while heating SiO_2_-IbuNa-M only to ca. 408 °C causes the main mass loss. The third stage of the mass reduction (by about 10% in the case of both of the mentioned samples) takes place at a higher temperature for SiO_2_-Nap-M, and the peak with a maximum at 596 °C is present on the DTG curve, while a maximum at 451 °C is observed on the DTG of SiO_2_-IbuNa-M.

**Figure A9:**
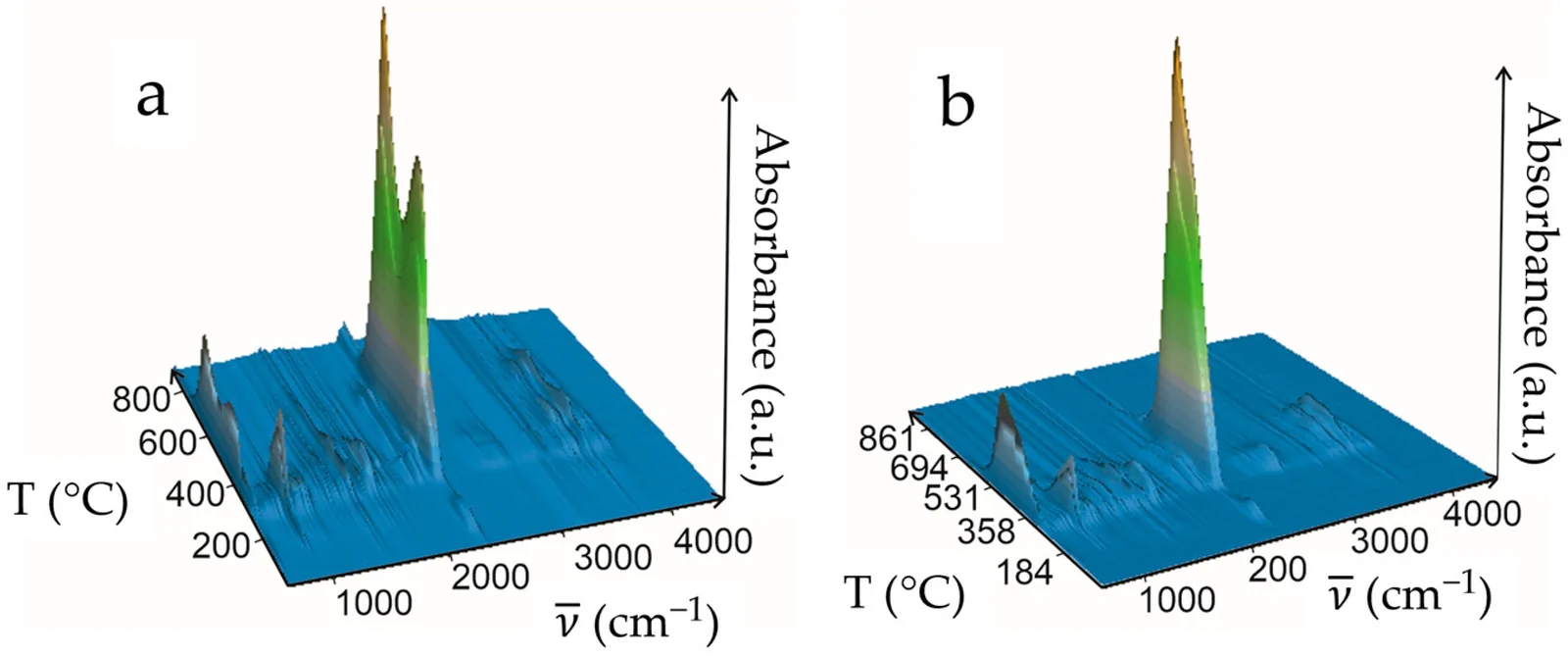
3D diagrams of FTIR spectra of volatiles formed during the heating of SiO_2_-Nap-M (**a**) and SiO_2_-IbuNa-M (**b**) in synthetic air. The colours represent different absorbance intensities.

**Figure A10:**
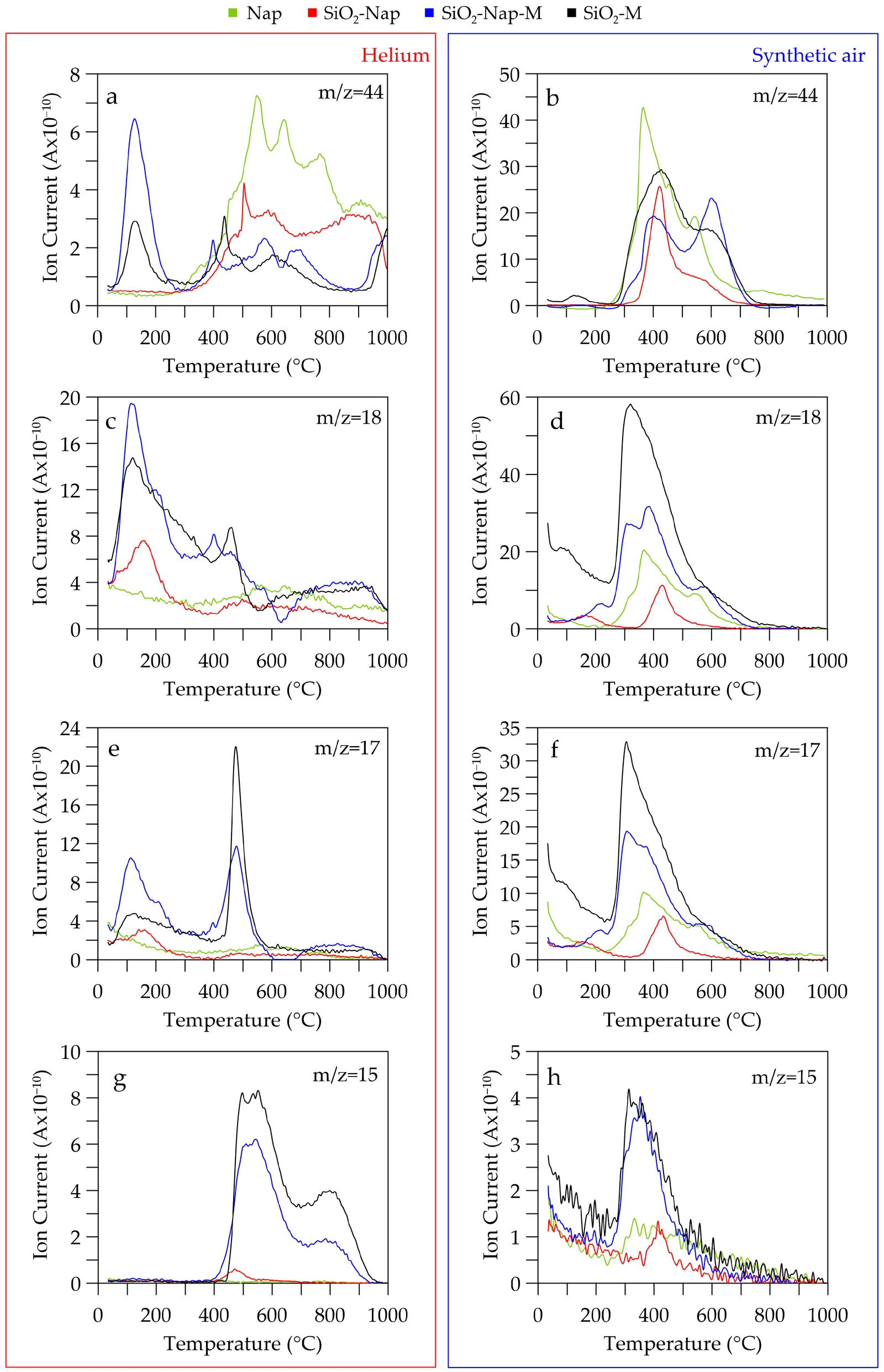
Temperature-dependent QMID (quasi-multiple ion detection) ion current profiles at selected *m*/*z* values, collected during the heating of investigated systems in helium (**a**,**c**,**e**,**g**) and in synthetic air (**b**,**d**,**f**,**h**). The coloured lines correspond to Nap (green), SiO_2_-Nap (red), SiO_2_-Nap-M (blue), and SiO_2_-M (black).

**Figure A11:**
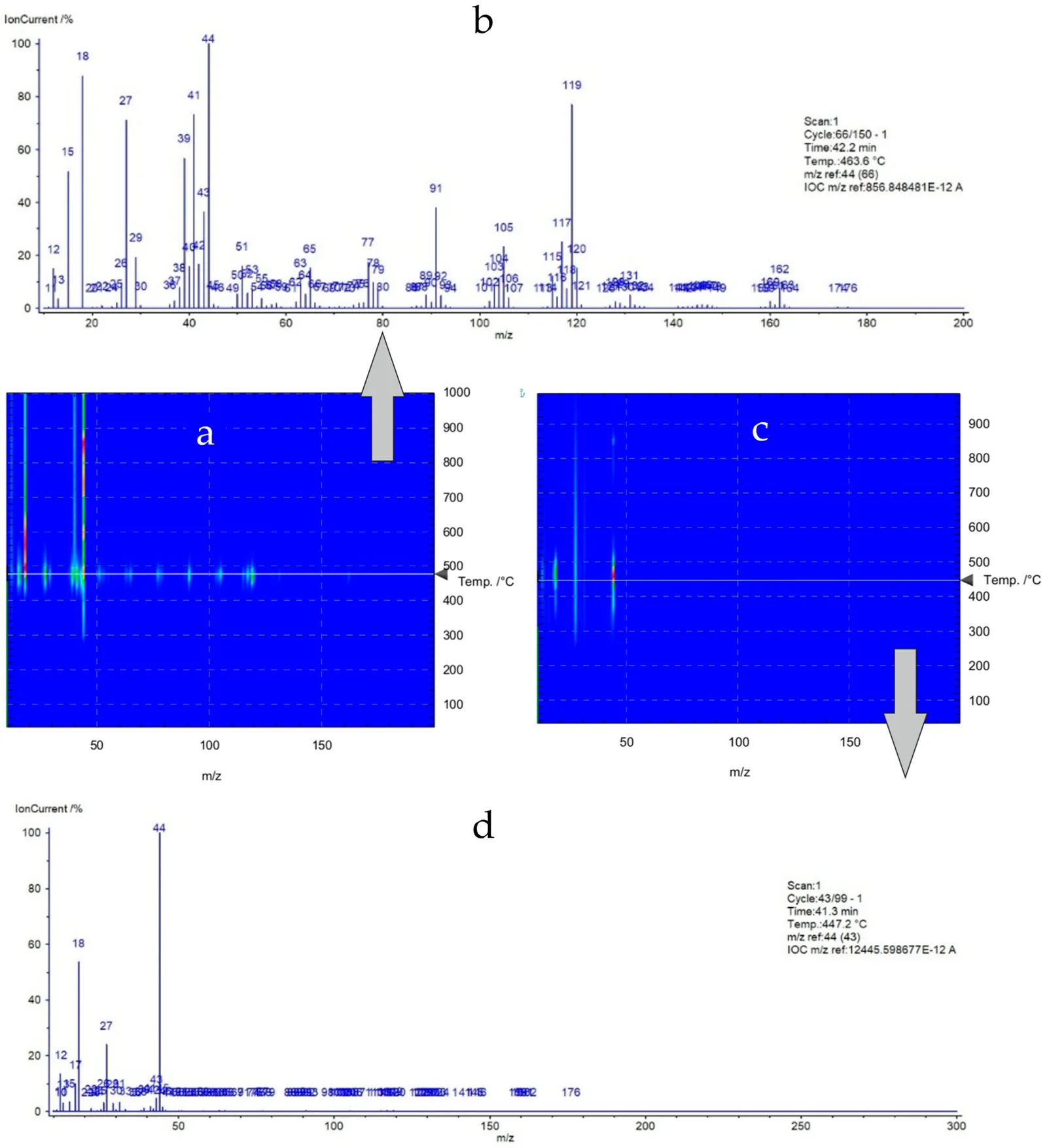
Temperature-dependent 2D plots of QMID (quasi-multiple ion detection) ion current curves collected during the heating of sodium ibuprofen in helium (**a**) and in synthetic air (**c**), and mass spectra of gases evolved from sodium ibuprofen detected at ca. 464 °C in helium (**b**) and at ca. 447 °C (**d**) in synthetic air. The colours in the 2D maps (**a,c**) indicate relative ion current (0–100%), increasing from blue (low) to red (high). The triangles on the right side of (**a,c**) indicate the temperatures at which the mass spectra shown in Figures (**b,d**) were recorded, respectively.

**Figure A12:**
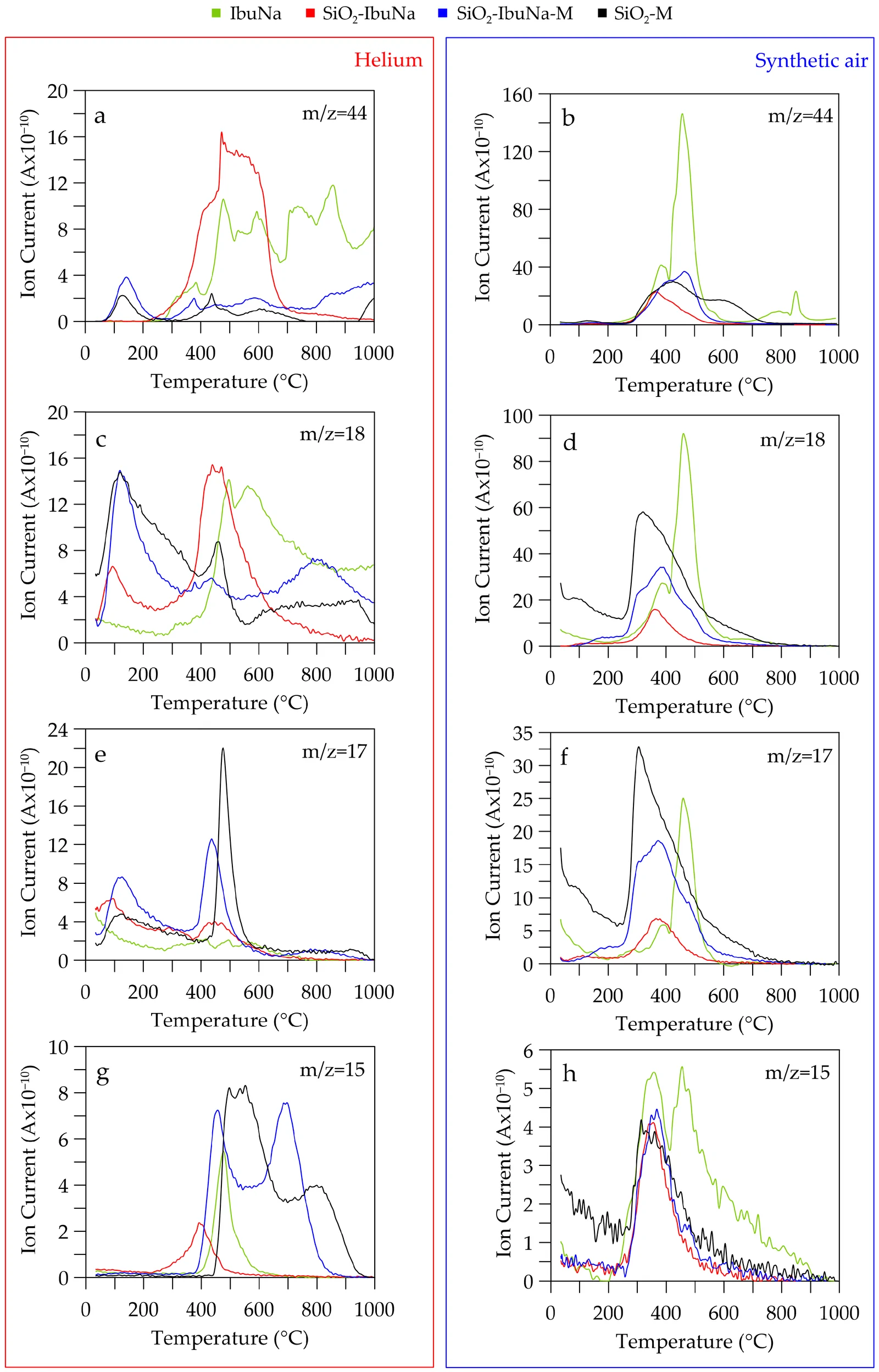
Temperature-dependent QMID (quasi-multiple ion detection) ion current profiles at selected *m*/*z* values, collected during the heating of investigated systems in helium (**a**,**c**,**e**,**g**) and in synthetic air (**b**,**d**,**f**,**h**). The coloured lines correspond to IbuNa (green), SiO_2_-IbuNa (red), SiO_2_-IbuNa-M (blue), and SiO_2_-M (black).

**Figure A13:**
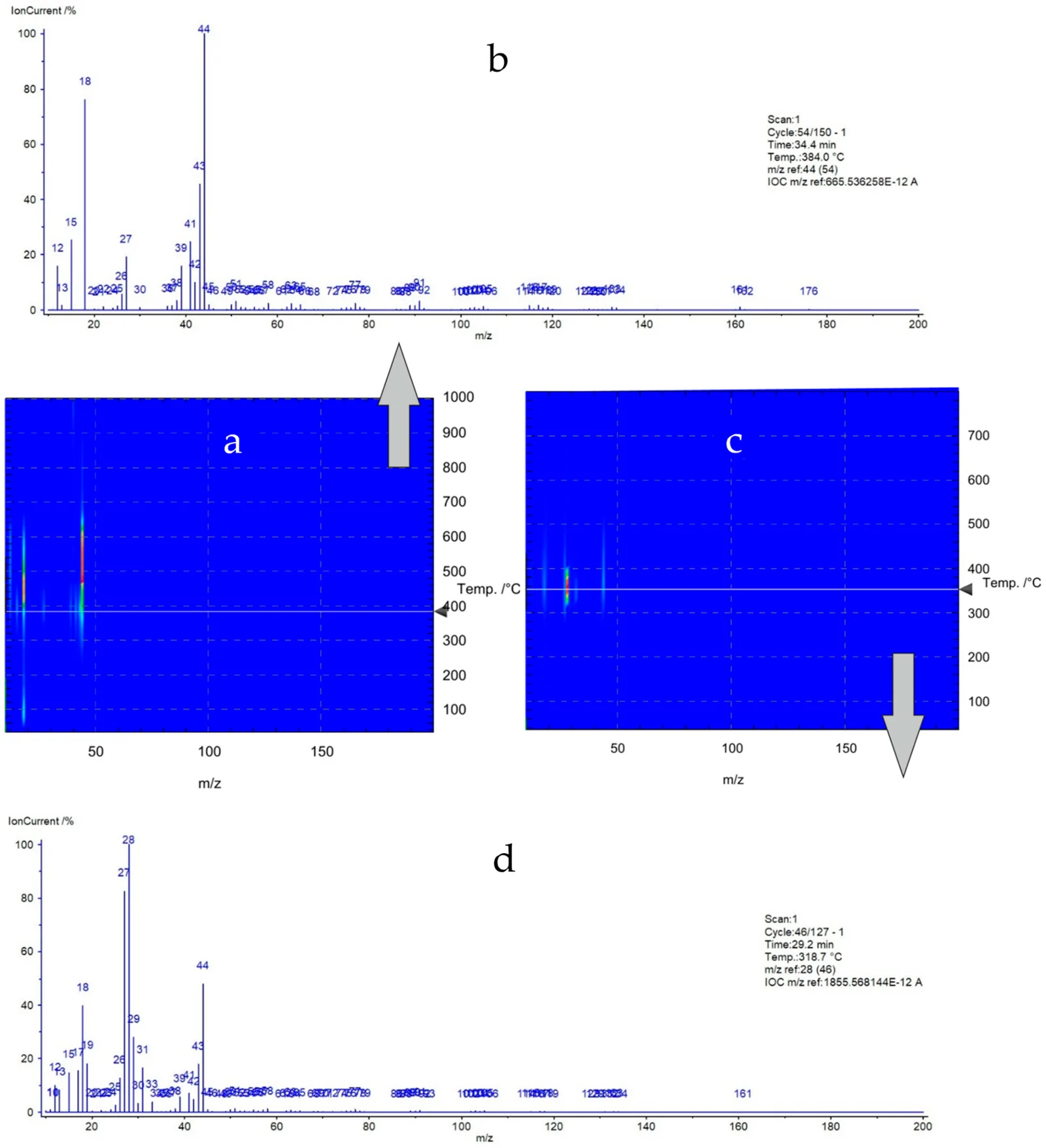
Temperature-dependent 2D plots of QMID (quasi-multiple ion detection) ion current curves collected during the heating of SiO_2_-IbuNa in helium (**a**) and in synthetic air (**c**), and mass spectra of gases evolved from SiO_2_-IbuNa detected at ca. 384 °C in helium (**b**) and at ca. 318 °C (**d**) in synthetic air. The colours in the 2D maps (**a,c**) indicate relative ion current (0–100%), increasing from blue (low) to red (high). The triangles on the right side of (**a**,**c**) indicate the temperatures at which the mass spectra shown in Figures (**b**,**d**) were recorded, respectively.

**Figure A14:**
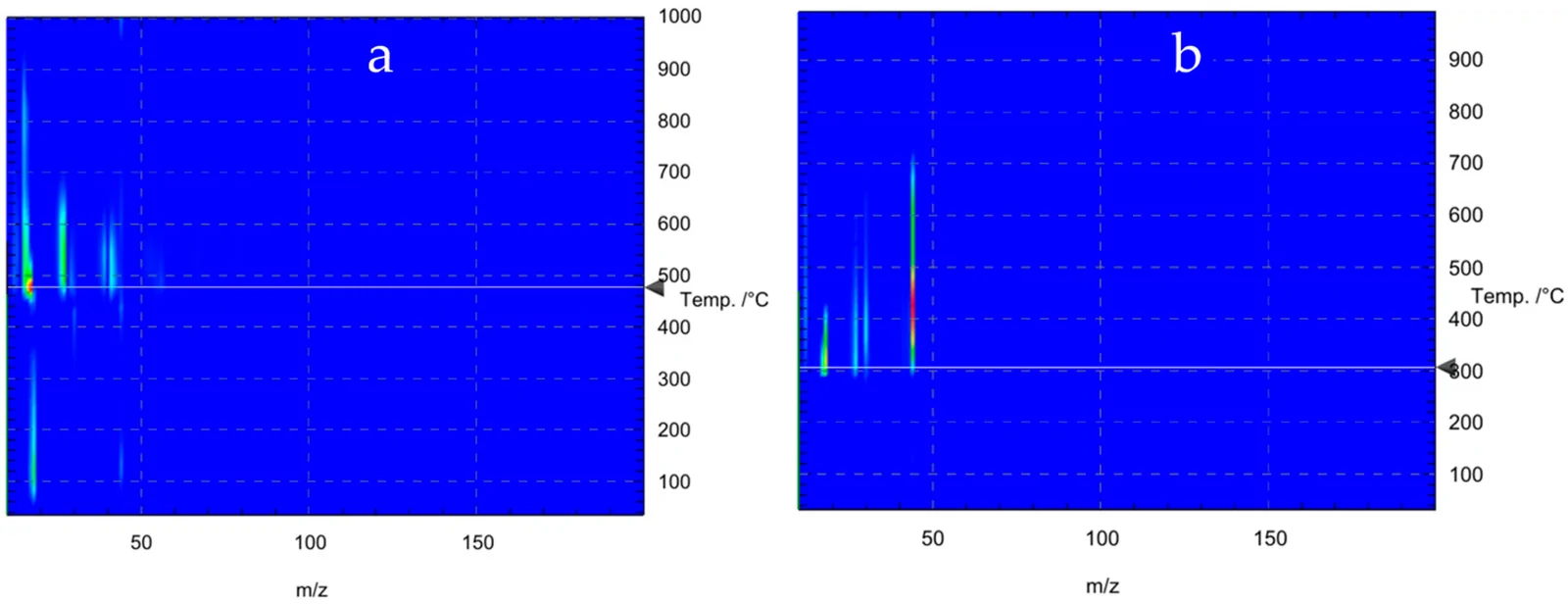
Temperature-dependent 2D plots of QMID (quasi-multiple ion detection) ion current curves collected during the heating of SiO_2_-M in helium (**a**) and in synthetic air (**b**). The colours in the 2D maps (**a,b**) indicate relative ion current (0–100%), increasing from blue (low) to red (high).

**Figure A15:**
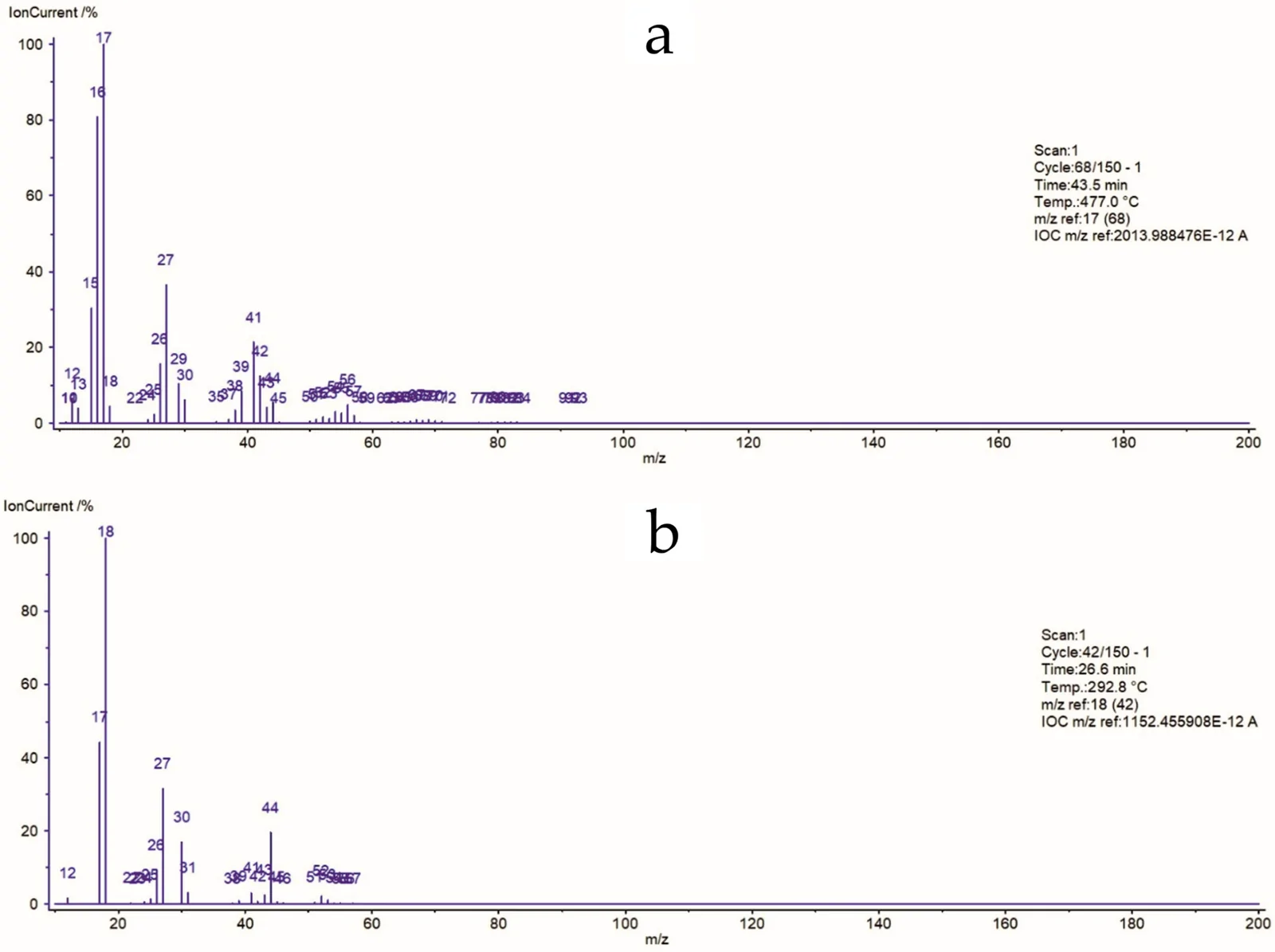
Mass spectra of gases evolved from SiO_2_-M detected at 477 °C in helium (**a**) and at ca. 293 °C (**b**) in synthetic air.

**Figure A16:**
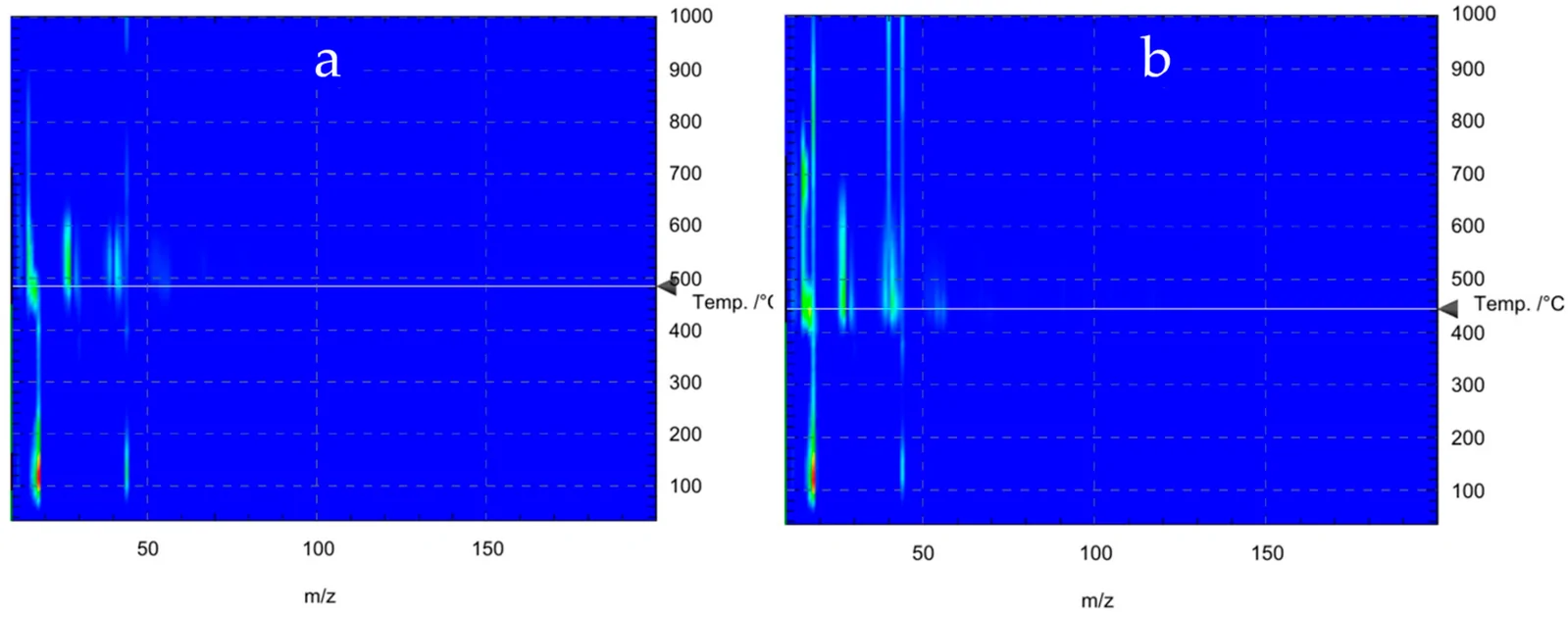
Temperature-dependent 2D plots of QMID ion current curves collected during the heating of SiO_2_-Nap-M (**a**) and SiO_2_-IbuNa-M (**b**) in helium. The colours in the 2D maps (**a,b**) indicate relative ion current (0–100%), increasing from blue (low) to red (high).

**Figure A17:**
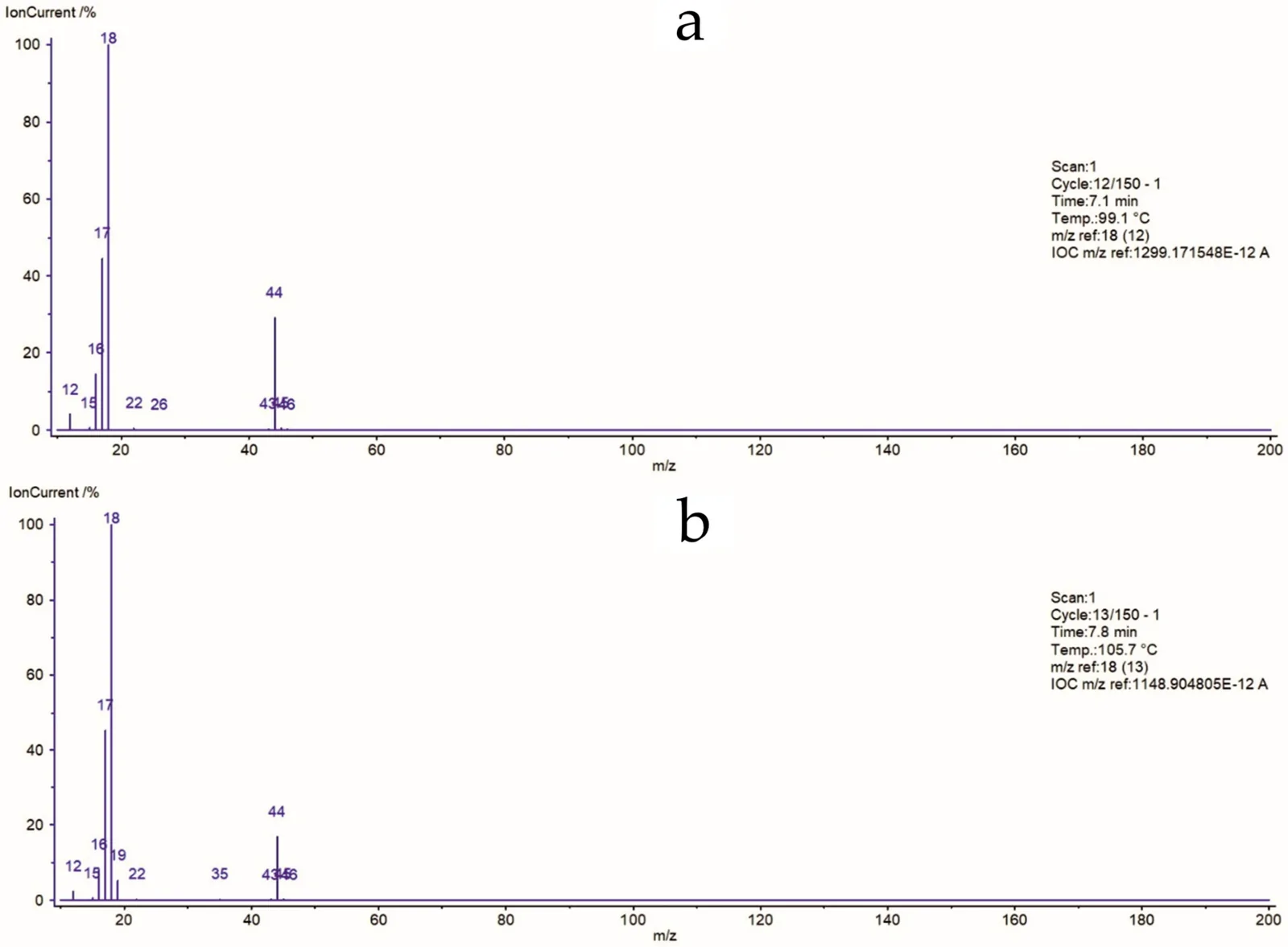
Scan mass spectrum of evolved gases from SiO_2_-Nap-M detected at 99 °C (**a**) and SiO_2_-IbuNa-M detected at 106 °C (**b**) in helium.

**Figure A18:**
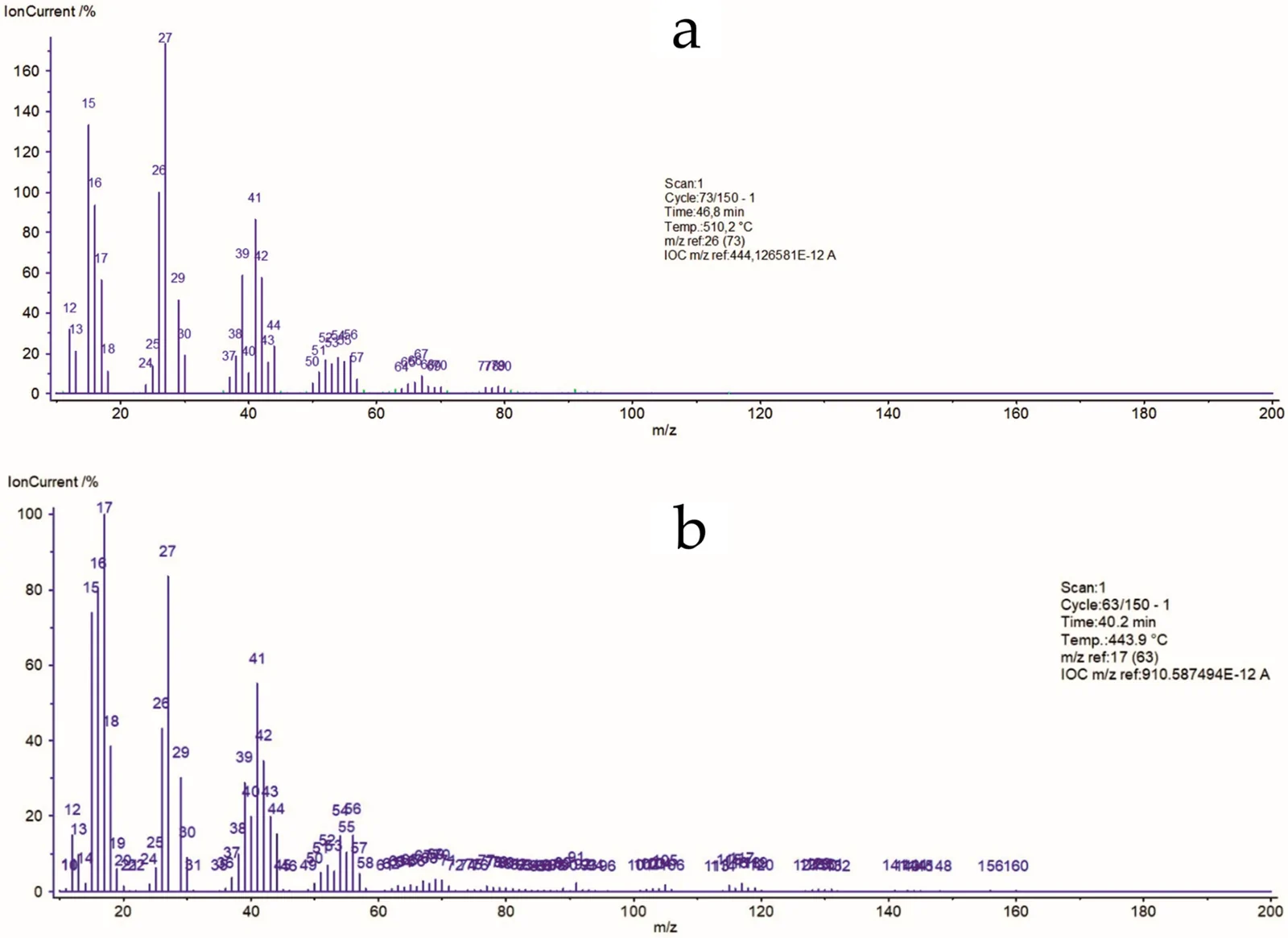
Scan mass spectrum of evolved gases from SiO_2_-Nap-M detected at 510 °C (**a**) and SiO_2_-IbuNa-M detected at 443.9 °C (**b**) in helium.

**Figure A19:**
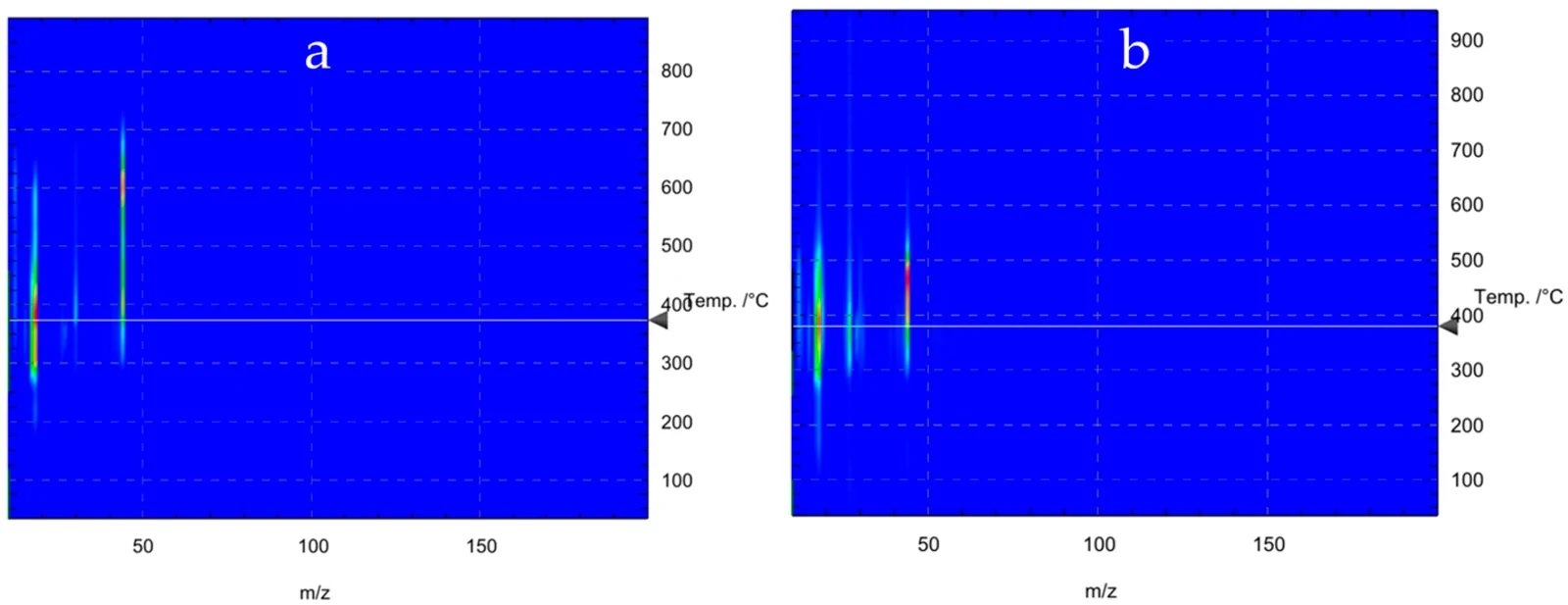
Temperature-dependent 2D plots of QMID (quasi-multiple ion detection) ion current curves collected during the heating of SiO_2_-Nap-M (**a**) and SiO_2_-IbuNa-M (**b**) in synthetic air. The colours in the 2D maps (**a,b**) indicate relative ion current (0–100%), increasing from blue (low) to red (high).

**Figure A20:**
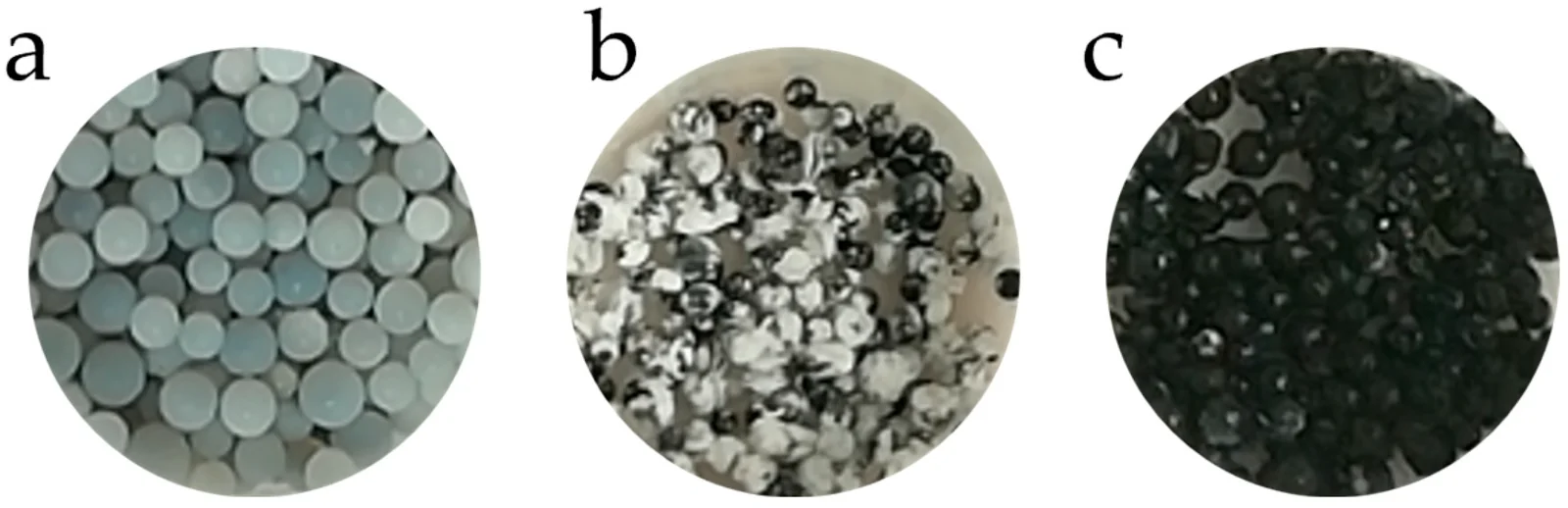
Representative image of SiO_2_-Nap (**a**), SiO_2_-IbuNa (**b**), and SiO_2_-M (**c**) remaining in the crucible after thermal analysis in helium.

**Figure A21:**
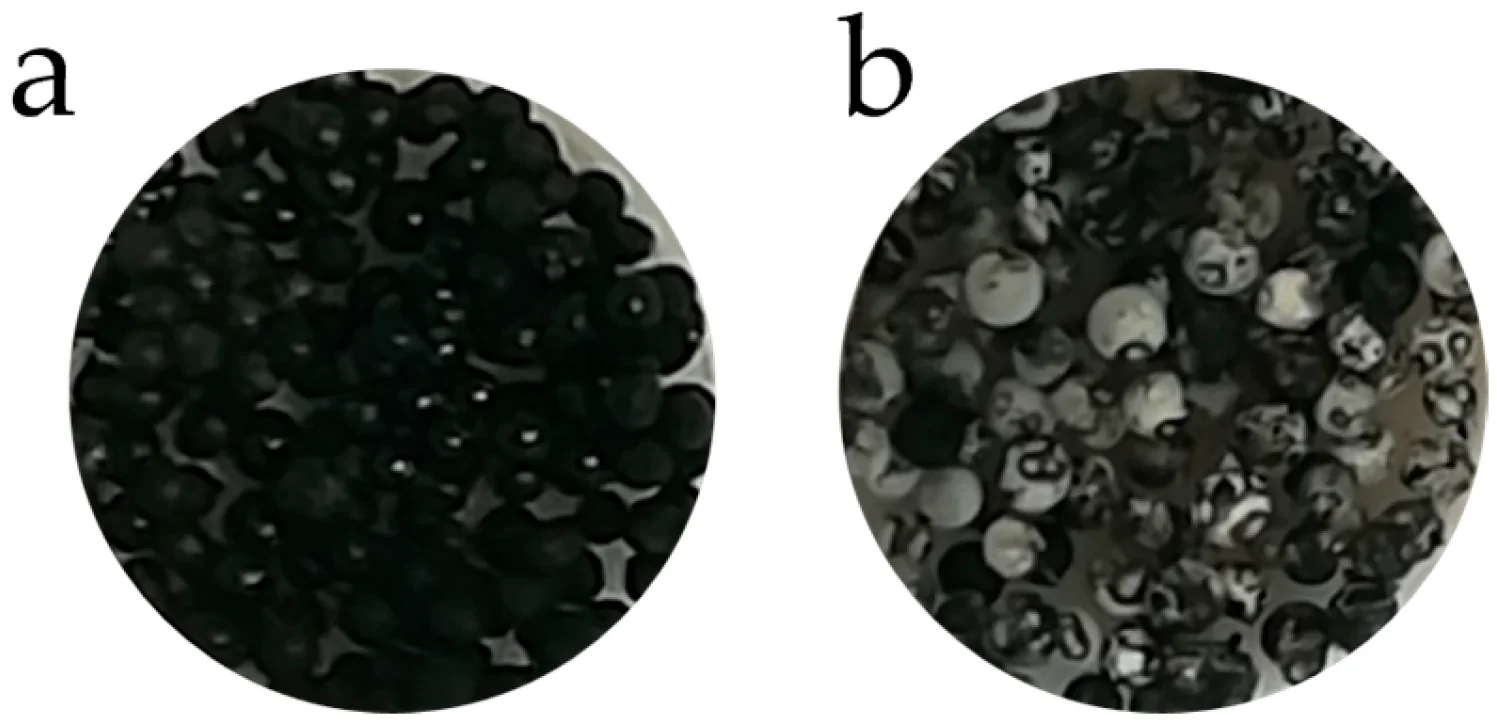
Representative image of SiO_2_-Nap-M (**a**) and SiO_2_-IbuNa-M (**b**) remaining in the crucible after thermal analysis in helium.

The DSC curves of SiO_2_-Nap-M and SiO_2_-IbuNa-M (Figure 7c,d) under oxidative conditions suggest a four-stage degradation process. The first event below 200 °C is endothermic, while the others are exothermic events. This endothermic event corresponds to the slight mass loss displayed by the TG curves and can be attributed to the desorption of water and NH_3_ (used for synthesis purposes), as evidenced by signals of *m*/*z* = 17 and 18 in the QMID spectra (Figure A19). Interestingly, the maxima of the first two exothermic peaks for both samples are detected at similar temperatures, i.e., approximately at 294 °C and 380 °C, while the third peak occurs at 459 °C for SiO_2_-IbuNa-M (Figure 7d) and only at 588 °C for SiO_2_-Nap-M (Figure 7c). The first two events (at lower temperatures) occur at roughly the same temperature for both drug-loaded samples as for SiO_2_-M, while the third falls within the same temperature range as for SiO_2_-M (Figure 5c) only for SiO_2_-Nap-M. From the analysis of MS profiles (Figure A19a) and 3D diagrams of FTIR spectra (Figure A9a) of the gaseous products emitted from drug-loaded samples, it appears that the oxidation profile of SiO_2_-Nap-M is similar to that of the SiO_2_-M sample (without naproxen), i.e., it starts with NH_3_ evolution from the decomposition of NH_2_ groups, followed by the CO and CO_2_ emission from the oxidation of the organic fraction. Similar findings were presented elsewhere [77]. Additionally, the course of SiO_2_-IbuNa-M oxidation is similar to that of SiO_2_-M, with the emission of similar volatile substances, except that it ends at a slightly higher temperature than 600 °C.

## 4. Conclusions

This study demonstrates the preparation of drug-loaded silica materials—in which porous silica pellets serve as the API carrier and amine-functionalized silica gel forms a functional NH_2_-SiO_2_ phase—and reports their thermal characterisation. SiO_2_ pellets synthesised via the hard-template method possessed an open porous architecture that facilitated the introduction of naproxen and sodium ibuprofen into their interior. On the other hand, the contribution from subnanometre free volumes in SiO_2_ was substantial and may correspond to closed micropore-like voids and/or free-volume regions associated with the thin silica walls separating neighbouring pores. Introducing either naproxen or sodium ibuprofen into SiO_2_ resulted in broadly similar changes in pore architecture, with preferential occupation of the smaller mesopores, while the remaining unoccupied mesopore network retained a considerable degree of connectivity. The changes observed by PALS in pore structure suggest the drug molecules were dispersed along the inner walls of the silica, although penetration into subnanometre voids could not be excluded. The formation of the amine-functionalized silica phase caused a pronounced decrease in both mesopore size and mesopore volume, with PALS analysis results suggesting that silica condensation was not restricted to the external pellet surface but also occurred throughout the accessible internal pore network, partially narrowing or filling micropore-scale free volumes. Moreover, the type of API dispersed in SiO_2_ affected the nanoscopic architecture of the resulting multicomponent systems.

The coupled TG–DSC–FTIR/MS analyses revealed pronounced atmosphere-dependent differences in the thermal decomposition of both investigated drugs. Naproxen decomposes endothermically in helium, involving the evolution of relatively high-molar-mass products, followed by their fragmentation at higher temperatures. The investigated sodium ibuprofen, which is substantially more thermally stable than naproxen, decomposed above 400 °C in helium, and the evolved gases produced a signal pattern indicative of 2-(4-isobutylphenyl)propionic acid (i.e., ibuprofen). Interestingly, in synthetic air where oxidative reactions predominate, the same pathway for sodium ibuprofen was also detected but to a much smaller extent.

The investigation of solid drug dispersions (in which PALS data suggested that drug molecules preferentially occupied smaller mesopores and were distributed along the silica walls) showed that silica markedly stabilised naproxen (by delaying its degradation in both gases rather than fundamentally changing its course) and reduced XRD-detectable crystallinity. The opposite effect was observed for sodium ibuprofen, which, immobilised in SiO_2_, became less thermally stable than the neat salt in both atmospheres. However, the course of decomposition of sodium ibuprofen immobilised in SiO_2_ in both atmospheres was similar to the neat drug. Differences in the number of decomposition steps between immobilised and free IbuNa suggested the presence of heterogeneous IbuNa populations in SiO_2_, possibly involving highly dispersed sodium ibuprofen on SiO_2_ and small clusters.

The H_2_O/NH_3_ vapour-assisted procedure enabled the formation of an amine-functionalized silica phase in all multicomponent systems. The PALS results suggest the NH_2_-SiO_2_ phase formation was not limited to the external SiO_2_-drug pellet surface but also involved the accessible pore network, thereby enhancing API confinement. In helium, this amine-functionalized silica phase increased the overall thermal stability of both drug-loaded systems. However, the decomposition of naproxen in the SiO_2_-Nap-M sample overlapped with that of the aminopropyl groups of the phase, while the principal decomposition pathway of sodium ibuprofen in the SiO_2_-IbuNa-M sample remained similar to that of the solid dispersion of IbuNa in SiO_2_, as signals compatible with volatile ibuprofen were detected together with H_2_O and CO_2_. This suggests that some sodium ibuprofen in the NH_2_-SiO_2_ phase in SiO_2_-IbuNa-M may have been located outside the silica pores or trapped within the hybrid phase. In contrast, in synthetic air, both multicomponent systems exhibited similar thermal behaviour, suggesting that oxidation was strongly influenced by the amine-functionalized silica phase and included desorption of water and residual NH_3_ (used for synthesis purposes), followed by degradation involving NH_3_ evolution from surface aminopropyl groups and subsequent oxidation of the organic fraction to mainly CO and CO_2_. In general, the oxidation process of the investigated systems containing the NH_2_-SiO_2_ phase is only slightly dependent on the selected drug.

## Figures and Tables

**Table 1:** Relative pore volume V of peaks with mean diameter D_mean_ in pore size distributions shown in Figure 1. Data for estimating the typical uncertainty are presented in Table A1, and the results in Table A2.

Diameter Range:	<0.5 nm	0.5–1.0 nm	1–2 nm	>8 nm
SiO_2_				
V (%)	17.1	37.2	6.4	39.3
D_mean_ (nm)	0.39	0.77	1.16	16.27
SiO_2_-IbuNa				
V (%)	20.6	17.0	1.1	61.4
D_mean_ (nm)	0.42	0.79	1.74	19.97
SiO_2_-Nap				
V (%)	17.0	22.2	2.0	58.8
D_mean_ (nm)	0.40	0.81	1.56	20.57
SiO_2_-IbuNa-M				
V (%)	63.2	29.3	–	7.5
D_mean_ (nm)	0.39	0.60	–	9.25
SiO_2_-Nap-M				
V (%)	74.9	16.7	–	8.4
D_mean_ (nm)	0.40	0.67	–	8.87

## Data Availability

The original contributions presented in this study are included in the article. Further inquiries can be directed to the corresponding author. Correspondence: Agnieszka Kierys: agnieszka.kierys@umcs.pl.

## Abbreviations

The following abbreviations are used in this manuscript:

API	active pharmaceutical ingredient
EU	European Union
TG	thermogravimetry
FTIR	Fourier-transform infrared spectroscopy
MS	mass spectrometry
DSC	differential scanning calorimetry
PALS	positron annihilation lifetime spectroscopy
MRI	magnetic resonance imaging
PET	positron emission tomography
CT	computed tomography
APTES	(3-aminopropyl)triethoxysilane
TEOS	tetraethoxysilane
SiO_2_	SiO_2_ pellets manufactured via hard template method
IbuNa	sodium ibuprofen
Nap	naproxen
EtOH	ethanol
LN_2_	low-temperature nitrogen adsorption/desorption
PSD	pore size distribution
BET	Brunauer−Emmett−Teller equation
BJH	Barrett−Joyner−Halenda equation
IUPAC	International Union of Pure and Applied Chemistry
QMID	quasi-multiple ion detection
XRD	X-ray diffraction

## Appendix B

### Low-Temperature Nitrogen Adsorption/Desorption (LN_2_)

Figure A2 shows the nitrogen adsorption and desorption isotherms for the SiO_2_ pellets obtained after burning out the Amberlite template. The adsorption isotherm appears to exhibit a combination of type II and type IV isotherms of the IUPAC classification [80]. Hence, SiO_2_ has pores of various sizes, from meso- to large macropores. Although the nitrogen adsorption and desorption isotherms coincide at low pressures, i.e., adsorption is completely reversible, a hysteresis loop is visible between the adsorption and desorption isotherms above approximately p/p_0_ = 0.7, the shape of which most closely resembles the H1 type, suggesting the presence of cylindrical pores. However, considering the synthesis conditions and previous reports [29,51], the presence of cylindrical pores in SiO_2_ prepared by the hard-template method using Amberlite^®^XAD7HP beads seems unlikely. Based on the previously presented TEM of silica gel prepared in the presence of water-ammonia vapour [29], the presence of channel-like or neck-like pores and non-uniform pore size distribution is more likely. Indeed, the pore size distribution is broad with a maximum at approximately 34 nm. The high pore volume (1.21 cm^3^/g) and high specific surface area (approximately 200 m^2^/g) indicate the relatively well-developed internal structure of the synthesised silica.

## Appendix F

### PALS Results Repeatability

**Table A1:** Relative pore volume V of peaks with mean diameter D_mean_ in pore size distributions obtained from subsequent measurements of silica pellets.

Diameter Range:	<0.5 nm	0.5–1.0 nm	1–2 nm	> 8 nm
SiO_2_				
V (%)	18.1	37.3	6.3	38.3
D_mean_ (nm)	0.39	0.77	1.19	16.90
SiO_2_				
V (%)	19.4	37.0	6.4	37.2
D_mean_ (nm)	0.34	0.76	1.17	16.09
SiO_2_				
V (%)	18.9	37.8	4.4	38.4
D_mean_ (nm)	0.39	0.79	1.26	16.4
SiO_2_				
V (%)	16.5	35.9	6.1	37.9
D_mean_ (nm)	0.39	0.77	1.17	16.27

**Table A2:** Average values and standard deviations (*σ*) of relative pore volumes (*V*) and mean diameters (D_mean_) presented in Table A1.

Diameter Range:	<0.5 nm	0.5–1.0 nm	1–2 nm	>8 nm
SiO_2_				
V (%)	18.2	37.0	5.8	38.0
*σV (%)*	*1.1*	*0.7*	*0.8*	*0.5*
D_mean_ (nm)	0.38	0.77	1.20	16.43
*σD_mean_ (nm)*	*0.02*	*0.01*	*0.04*	*0.30*
